# Oxygen Anion Redox Chemistry Correlated with Spin State in Ni‐Rich Layered Cathodes

**DOI:** 10.1002/advs.202206442

**Published:** 2023-01-25

**Authors:** Zhihua Lu, Jicheng Zhang, Qinghua Zhang, Deniz Wong, Wen Yin, Nian Zhang, Zhongjun Chen, Lin Gu, Zhongbo Hu, Xiangfeng Liu

**Affiliations:** ^1^ Center of Materials Science and Optoelectronics Engineering College of Materials Science and Optoelectronic Technology University of Chinese Academy of Sciences Beijing 100049 P. R. China; ^2^ Beijing National Laboratory for Condensed Matter Physics Institute of Physics Chinese Academy of Science Beijing 100190 P. R. China; ^3^ Dynamics and Transport in Quantum Materials Helmholtz‐Zentrum Berlin für Materialen und Energie Albert‐Einstein‐Strasse 15 12489 Berlin Germany; ^4^ Spallation Neutron Source Science Center Dongguan 523803 P. R. China; ^5^ Shanghai Institute of Microsystem and Information Technology Chinese Academy of Sciences Shanghai 200050 P. R. China; ^6^ Lirong Zheng Beijing Synchrotron Radiation Facility Institute of High Energy Physics Chinese Academy of Sciences Beijing 100049 China; ^7^ CAS Center for Excellence in Topological Quantum Computation University of Chinese Academy of Sciences Beijing 100190 China

**Keywords:** chemo‐mechanical heterogeneity, Li/Ni antisite defects, Ni‐rich layered oxides, oxygen redox, spin state

## Abstract

Despite the low cost and high capacity of Ni‐rich layered oxides (NRLOs), their widespread implementation in electric vehicles is hindered by capacity decay and O release. These issues originate from chemo‐mechanical heterogeneity, which is mainly related to oxygen anion redox (OAR). However, what to tune regarding OAR in NRLOs and how to tune it remains unknown. In this study, a close correlation between the OAR chemistry and Li/Ni antisite defects is revealed. Experiments and calculations show the opposite effects of aggregative and dispersive Li/Ni antisite defects on the NiO_6_ configuration and Ni spin state in NRLOs. The resulting broad or narrow spans for the energy bands caused by spin states lead to different OAR chemistries. By tuning the Li/Ni antisite defects to be dispersive rather than aggregative, the threshold voltage for triggering OAR is obviously elevated, and the generation of bulk‐O_2_‐like species and O_2_ release at phase transition nodes is fundamentally restrained. The OAR is regulated from irreversible to reversible, fundamentally addressing structural degradation and heterogeneity. This study reveals the interaction of the Li/Ni antisite defect/OAR chemistry/chemo‐mechanical heterogeneity and presents some insights into the design of high‐performance NRLO cathodes.

## Graphical abstract

The aspects of oxygen anion redox (OAR) that should be tuned in Ni‐rich cathodes for improved performance and means of doing so is not clearly elucidated. Herein, a basic factor of spin state that influences OAR is discovered. Distinct spin states result from aggregative and dispersive Li/Ni disorder. The mechanism of spin states in tuning OAR chemistry is revealed.

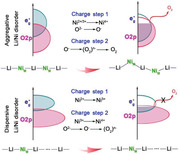

## 1 Introduction

The widespread implementation of electric vehicles (EVs) is essential for decarbonization, which presently calls for advanced cathodes with low cost and high capacity. Ni‐rich transition metal (TM) layered oxides (LiNi*_x_* Co*_y_* Mn_1−_ *_x_*_−_ *_y_* O_2_, *x* > 0.7) are among the most promising cathodes for Li‐ion batteries and have shown great potential for use in EVs.^[^ ^1^, ^2^ ^]^ A high Ni content enables LiNi*_x_* Co*_y_* Mn_1−_ *_x_*_−_ *_y_* O_2_ to have a high specific capacity and a low proportion of expensive and toxic Co. However, the critical issues of rapid capacity decay and safety concerns plague this series of cathodes.^[^ ^3^ ^]^ Following extensive studies, a consensus has been reached that the chemo‐mechanical heterogeneity of grains accounts for electrode failure. Surface reconstruction, uneven Li depletion, intragranular nanopores, and internal void spaces were observed in the oxides.^[^ ^4^, ^5^, ^6^, ^7^, ^8^ ^]^ Grey et al. revealed that the lattice strain between the reconstructed surface and bulk layered structure induces the fatigue process of the cathode.^[^ ^9^ ^]^ Furthermore, the heterogeneity of Ni‐rich layered oxides originates from the inhomogeneous chemical reactions in the cathodes. Efforts have been made to perform surface modification and bulk doping, as well as to realize single‐crystalline particles.^[^ ^10^, ^11^, ^12^, ^13^, ^14^ ^]^ Although improvements have been achieved to some extent, the practical electrode performance remains far from satisfactory for large‐scale commercialization, and the underlying mechanism for addressing the heterogeneity and degradation of LiNi*_x_* Co*_y_* Mn_1−_ *_x_*_−_ *_y_* O_2_ cathodes remains to be clearly elucidated.

With increasing research on oxygen anion redox (OAR), correlations between the complex OAR behaviors and cathode heterogeneity have been proven. Issues of O release and phase degradation, spreading from the surface to the bulk of the cathode grains, initiate cathode heterogeneity.^[^ ^5^, ^15^, ^16^, ^17^ ^]^ The fundamental tuning of the OAR chemistry is crucial for eliminating the chemo‐mechanical heterogeneity of Ni‐rich cathodes. However, this task is difficult because a complete understanding of the relationship between the structural characteristics and the corresponding lattice O reactions remains elusive. In particular, the factors that trigger OAR in Ni‐rich oxide cathodes are poorly understood. It is necessary to reveal the structural factors that influence the OAR chemistry in Ni‐rich cathodes and tune the OAR reversibility to address the capacity decline and safety concerns.

Li/Ni antisite defects are basic and unavoidable structural defects in LiNi*_x_* Co*_y_* Mn_1−_ *_x_*_−_ *_y_* O_2_. Pristine and cycled Ni‐rich cathodes have been proven to exhibit Li/Ni antisite disorders. Extensive research on Li/Ni antisite defects has been conducted, and the detrimental and beneficial effects of Li/Ni disorder have recently been debated.^[^ ^18^, ^19^, ^20^, ^21^ ^]^ The different effects may be due to the different characteristics of the Li/Ni antisite defects, which were overlooked in these works. Different Li/Ni disorders should have varied effects on the local crystal and electronic structure near the defects, and the overall structural properties of the material will also change accordingly.^[^ ^22^, ^23^ ^]^ Previous studies have shown that the microstructure plays an important role in OAR chemistry through band structure regulation.^[^ ^24^, ^25^, ^26^ ^]^ Obviously, Li/Ni antisite defects with specific characteristics should exhibit an influence. However, the relationship between Li/Ni antisite defects with different local structures and the OAR reaction path/reaction rate/reversibility remains unclear. Therefore, it is important to explore the influence mechanisms of specific Li/Ni mixing on the OAR chemistry to realize a fundamental solution for chemo‐mechanical heterogeneity.

In this study, LiNi_0.8_Co_0.1_Mn_0.1_O_2_ with characteristics of aggregative or dispersive Li/Ni antisite defects was designed and synthesized. The OAR was tuned from irreversible to reversible by changing the spin states of the Li/Ni antisite defects. By utilizing both experimental and calculation techniques, the fundamental correlations between the OAR chemistry and Li/Ni antisite defects were fully elucidated for the first time. The aggregative Li/Ni antisite defect shows an elevated Ni spin state and an expanded span for the energy bands. The elevated Ni spin state quickens Ni oxidation and compels some electrons in the O2p band to participate in charge compensation with delithiation. Abundant bulk‐O_2_‐like species are generated in the lattice at nodes for phase changes of H1→M, M→H2, and H2→H3 (H: hexagonal, M: monoclinic) with simultaneous O_2_ gas release and remarkable NiO_6_ distortions, which contribute to severe heterogeneity. In contrast, Ni exhibits a relatively low spin state in dispersive Li/Ni antisite‐defected LiNi_0.8_Co_0.1_Mn_0.1_O_2_. O oxidation is largely inhibited by producing only a small amount of (O_2_)*^n^* ^−^. After discharge, (O_2_)*^n^* ^−^ was completely reduced to lattice O^2−^, whereas the bulk‐O_2_‐like species were only partly reduced to O^−^. Thus, OAR reversibility and heterogeneity were fundamentally improved. The modulated cathode showed enhanced electrochemical kinetics, a high capacity at 0.1 C (>200 mAh g^−1^), and good capacity retention at 0.2 C (93.8%@100 cycles). Some insights into the interaction between Li/Ni antisite defect/OAR chemistry/chemo‐mechanical heterogeneity were revealed, shedding light on OAR tuning for high‐performance Ni‐rich cathodes.

## 2 Results and Discussion

### 2.1 Constructing Aggregative and Dispersive Li/Ni Antisite Defects

The aggregative Li/Ni‐disordered and dispersive Li/Ni‐disordered LiNi_0.8_Co_0.1_Mn_0.1_O_2_ cathodes are labeled 811‐A and 811‐D, respectively. Precursors with different Ni distributions were designed to synthesize the two cathodes. A schematic of the synthesis process is provided in Figure S1 (Supporting Information). Here, we lowered the relative Ni content on the surface of the precursor for 811‐A by H^+^ etching and elevated the relative Ni content on the surface of the precursor for 811‐D by Ni reprecipitation. Scanning electron microscopy (SEM) images showed that the morphologies of the two precursors were almost identical (Figure S2, Supporting Information). Combined with Ar^+^ etching, X‐ray photoemission spectroscopy (XPS) was used to probe the compositions of the precursor surfaces. The results showed that the prepared precursors conformed to our design (Figure S3, Supporting Information). After heat treatment at 150 °C, much more metal Ni is yielded in the precursor for 811‐A than 811‐D (Figure S4, Supporting Information). Owing to the densely packed atomic arrangement of Ni in the metal state, these Ni atoms must overcome the migration energy to form a layered oxide. Some of the atoms that failed to migrate eventually formed mixed Ni cations. Because of the higher content of Ni metal, more mixed Ni cations were formed in 811‐A than in 811‐D after sintering, resulting in aggregated Li/Ni disorder for 811‐A and dispersive Li/Ni disorder for 811‐D.

**Figure 1:**
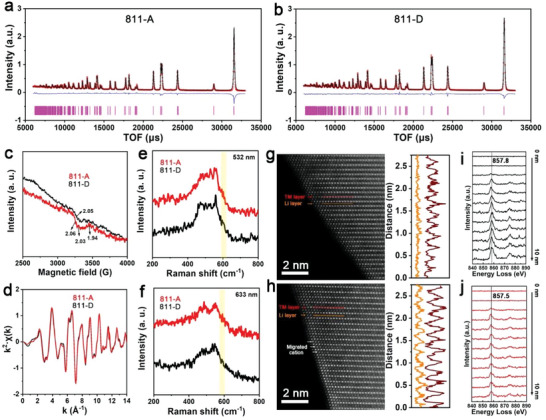
a,b) Rietveld refinement of neutron diffraction profiles of 811‐A and 811‐D. c) EPR spectra of 811‐A and 811‐D at 4 K. d) Raw Ni EXAFS results in the k‐space of 0–14 Å^−1^. e,f) Raman profiles of 811‐A and 811‐D with wavelengths of 532 and 633 nm. g,h) AC‐STEM images and line intensities of 811‐A and 811‐D. i,j) Ni L2,3‐edge EELS results for 811‐D and 811‐A. TM: transition metal.

Time‐of‐flight secondary ion mass spectrometry depth profiles revealed various NiO_2_ signals on the surfaces of the prepared cathodes (Figure S5, Supporting Information). Inductively coupled plasma (ICP) optical emission spectrometry demonstrated a close contrast between the TM elements in 811‐A and 811‐D (Table S1, Supporting Information). These results indicate that the differences in the elemental compositions of the materials are on the surface. The morphologies of 811‐A and 811‐D are similar, as evidenced by the SEM images in Figure S6 (Supporting Information). The peak splitting of the 006, 012 pair and 018, 110 pair in the X‐ray diffraction (XRD) plots for 811‐A and 811‐D (Figure S7, Supporting Information) indicate a well layered *α*‐NaFeO_2_ structure.^[^ ^27^ ^]^ The Rietveld refinement diagrams and results are provided in Figure S8 and Table S2 (Supporting Information). Neutron diffraction (ND) accompanied by Rietveld refinement of 811‐A (**Figure** **1**a) and 811‐D (Figure 1b) was performed. The metal ratio for refinement was based on the ICP results. Tables S3 and S4 (Supporting Information) list the refinement results, demonstrating that the refined XRD and ND results are consistent. Compared to those of 811‐D, the crystal cell parameters *a* and *c* of 811‐A are lower. The average contents of Li/Ni antisite defect in 811‐A and 811‐D are 5.18(8)% and 2.39(4)%, respectively. O vacancies appeared in 811‐A but not in 811‐D. This finding is supported by another report that a high Li/Ni antisite defect ratio induces the formation of O defects.^[^ ^22^ ^]^ The XPS results indicate that the O vacancies were mainly located on the surface of 811‐A, as demonstrated by the shifts in the depth spectra (Figure S9, Supporting Information).

O vacancies accompanied by Li/Ni antisite defects change the coordination of the TM cations, including a high TM spin state.^[^ ^28^ ^]^ The more complicated single‐electron state for 811‐A than 811‐D, as determined by electron paramagnetic resonance (EPR) testing at 4 K, verifies this point (Figure 1c). As a result of the high spin state, a larger energy span of electron‐occupied states for 811‐A than for 811‐D is exhibited, as evidenced by the XPS valence band spectra (Figure S10, Supporting Information). The average bulk structural properties of both the materials are similar, as demonstrated by the similar raw extended X‐ray absorption fine structure (EXAFS) data for Ni, Co, and Mn (Figure 1d and Figure S11, Supporting Information).

Raman spectroscopy with different lasers was used to explore 811‐A and 811‐D further at various depths (Figure 1e,f). The detection depth of the 532 nm laser is ≈50 nm, which is half that of the 633 nm laser. Two vibration peaks attributed to O—Ni(Co/Mn)—O bending (*E*_g_) and Ni(Co/Mn)—O stretching (*A*_1g_) were observed at Raman shifts of ≈500 and 550 cm^−1^, respectively.^[^ ^29^ ^]^ In the LiNi*_x_* Co*_y_* Mn_1−_ *_x_*_−_ *_y_* O_2_ cathodes, these two peaks widen because of the slightly different peak positions for different TM elements. In Figure 1e (532 nm), compared with 811‐L, 811‐H exhibits a spectrum with a slight move to a lower Raman shift, which is explained by the lower response wave number for Ni than for Mn (or Co). Meanwhile, at ≈600 cm^−1^, a peak representing a cation antisite defect was distinctly observed in the spectrum of 811‐A but not in that of 811‐D.^[^ ^30^ ^]^ In Figure 1f (633 nm), the differences between the two samples shown in Figure 1e are significantly weakened, demonstrating that a large number of aggregated Li/Ni antisite defects appeared on the 811‐A surface but not on the 811‐D surface.

The surface structural features of the two materials were explored using spherical aberration‐corrected scanning transmission electron microscopy (AC‐STEM). The bright‐spot array represents the TM layer, whereas the interlayer with a dark appearance represents the Li layer. For 811‐D (Figure 1g), the full darkness and line intensity in the Li layer indicate the rare occurrence of TM cation migration. For 811‐A (Figure 1h), a large number of bright spots are observed in the interlayers, which signify abundant migrated TM cations. Moreover, the ordered arrangement of the migrated cations is reflected by the regular appearance of peaks in the line intensity in the Li layer. By calculating the ratio of the peak area for the line intensity in the Li layer to that for the line intensity in the TM layer, Li/Ni mixed contents of 811‐A and 811‐D in the corresponding regions were quantified to be 29.5% and 4.3%, respectively. The shift of the L3 peak for Ni from 857.8 (811‐D) to 857.5 eV (811‐A) in the electron energy loss spectroscopy (EELS) spectra (Figure 1i,j) indicates an elevated spin state for 811‐A. An increasing layer spacing from the inner to the surface in 811‐A was also detected (Figure S12, Supporting Information), which was induced by inhomogeneous states. These results illustrate an aggregative Li/Ni antisite defect in 811‐A and a dispersive Li/Ni antisite defect in 811‐D.

### 2.2 Electrochemical Performance

**Figure 2:**
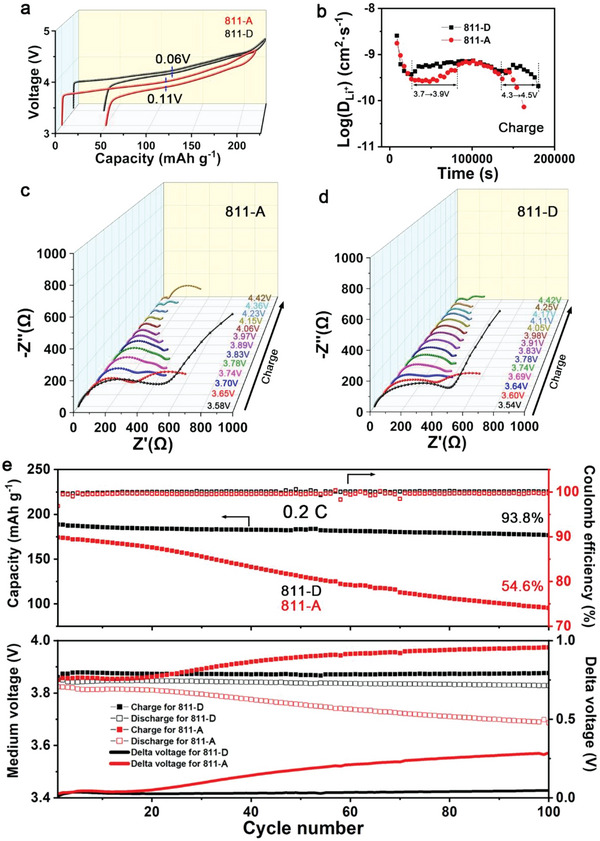
a) Initial charge–discharge plots. b) Calculated Li^+^ diffusion coefficient for the charging process from GITT. c,d) In situ EIS plots of 811‐A and 811‐D at different voltages for the charging process. e) Cycling performances of 811‐D and 811‐A at 1 C, where 1 C was set as 200 mA g^−1^. The delta voltage is the difference between the medium voltages for charging and discharging.

The charge–discharge plots of the initial cycle show a greater voltage hysteresis for 811‐A than for 811‐D (**Figure** **2**a), and the *d*Q/*d*V curves indicate the differences in the redox peaks of the two materials (Figure S13, Supporting Information). In contrast to the independent oxidation peaks in 811‐D, a certain degree of overlap for peaks denoting H1→M and M→H2 transitions is observed in 811‐A. In addition, an extra peak at ≈4.5 V is observed in 811‐A, which may be related to the OAR. The galvanostatic intermittent titration technique (GITT) revealed different redox mechanisms for the two samples (Figure 2b and Figure S14, Supporting Information). The Li^+^ diffusion coefficient (*D*_Li_^+^) for the two samples is at the same level in most electrochemical states, except in the charge voltage intervals of 3.7–3.9 and 4.3–4.5 V. This change in *D*_Li_^+^ was related to the different redox behaviors of the two materials. Electrochemical impedance spectroscopy (EIS) was additionally performed to explore the impedance (Figure 2c). The phase transition of the electrode material increases the semicircle number by generating solid–solid interfaces.^[^ ^31^ ^]^ During the early charging stage, both cathodes changed from a single interface to a double interface, as determined by the number of semicircles in the EIS plots. For 811‐D, after charging to 3.78 V, the two semicircles merge into one. With further charging to 4.05 V, two semicircles appear again. These changes demonstrate the typical phase transitions H1→M, M→H2, and H2→H3. In contrast, 811‐A maintains two semicircles throughout the charging process, which signifies that some components of 811‐A influence phase transitions.

The rate capability of 811‐D is better than that of 811‐A (Figure S15, Supporting Information). The charge–discharge curves at different rates and the corresponding *d*Q/*d*V plots exhibit a smaller change from 0.1 to 10 C for 811‐D than for 811‐A (Figure S16, Supporting Information), indicating faster electrochemical kinetics for 811‐D. Upon cycling at 0.2 C, the capacity–voltage plots and *d*Q/*d*V curves indicate that the oxidation/reduction peaks decreased rapidly in 811‐A but remained almost constant in 811‐D (Figure S17, Supporting Information). As Figure 2d shows, 811‐D has a capacity retention of 93.8% after cycling at 0.2 C, much higher than the value of 54.6% for 811‐A. The average Coulombic efficiency (2–100 cycles) of 811‐D at 0.2 C is almost 100%, which is higher than that of 811‐A (99.6%). Meanwhile, 811‐A demonstrates rapid changes in medium voltage values for charge–discharge curves and their delta voltage values, which are nearly unchanged for 811‐D. The cycling at 1C is also much more stable for 811‐D than for 811‐A (Figure S18, Supporting Information). These electrochemical properties indicate that compared with 811‐D, 811‐A has slower redox kinetics, more complex interfacial structural changes, and more unstable redox behaviors, which are closely related to the redox mechanism and structural evolution.

### 2.3 Distinct OAR Chemistries

**Figure 3:**
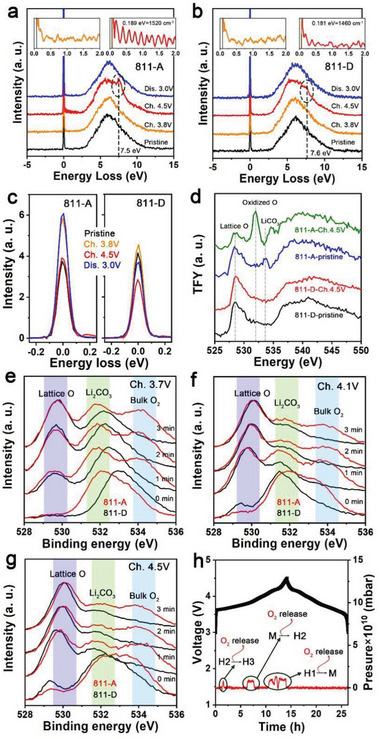
a,b) Normalized O–K‐edge RIXS for 811‐A and 811‐D electrodes. The insets indicate the O—O vibration peaks. c) Elastic peaks of normalized O–K edge RIXS spectra. d) O–K‐edge spectra for pristine and 4.5 V charged electrodes in TFY modes. e–g) O 2p XPS depth spectra for Ch. 3.7 V, Ch. 4.1 V, and Ch. 4.5 V electrodes. h) Operando DEMS results for 811‐A. Ch. stands for charge, and Dis. stands for discharge.

O–K‐edge resonant inelastic X‐ray scattering (RIXS) spectra were used to elucidate the O redox reactions of 811‐A and 811‐D (**Figure** **3**a,b), using an excitation energy of 531 eV.^[^ ^32^ ^]^ All RIXS spectra were normalized based on the inelastic peak at an energy loss of ≈6 eV (corresponding to ≈525 eV). In the pristine state, the inelastic peak of 811‐A is wider than that of 811‐D, which implies an enlarged energy band span for 811‐A (Figure S19, Supporting Information). In the 4.5 V charged state, the inelastic peaks at an energy loss of 7.5 eV (≈523.5 eV in absolute energy) for both materials exhibit obvious differences. The inelastic peak at 7.5 eV is considered to be the fingerprint of the lattice O redox.^[^ ^33^, ^34^ ^]^ Compared with 811‐A, the lattice O redox in 811‐D is significantly inhibited, as evidenced by the intensities of the O redox peaks. Moreover, the OAR fingerprint is located at a lower energy loss for 811‐A than for 811‐D, as shown in Figure S20 (Supporting Information), indicating a higher electron‐occupied state for the oxidized O species in 811‐A than in 811‐D, which reflects a deeper O oxidation in 811‐A than in 811‐D.

The vibrational peaks near 0 eV are caused by the O—O interactions. The amplitudes and frequencies of the vibration peaks convey information about the content and character of the O‐oxidized species.^[^ ^24^, ^35^ ^]^ As shown in Figure 3a,b, the 811‐A and 811‐D electrodes do not exhibit vibration peaks until the 4.5 V charged state. The higher peak amplitude for 811‐A than for 811‐D illustrates that more substances with O—O interactions are produced by 811‐A, which comes from a higher OAR activity. Different vibration frequencies are exhibited by materials that reflect different O—O distances.^[^ ^24^ ^]^ A large vibrational frequency implies a strong O—O interaction, which reflects a short O—O distance. The vibrational frequency for 4.5 V charged 811‐A is 0.189 eV (corresponding to 1520 cm^−1^), whereas that for 4.5 V charged 811‐D is 0.181 eV (corresponding to 1460 cm^−1^). The O—O interactions and shorter O—O distances of the O oxidized species for 811‐A than for 811‐D are demonstrated by these values. The different vibrational frequencies were verified using Raman spectroscopy (Figure S21, Supporting Information), which detected the 1520 cm^−1^ signal in 811‐A but not in 811‐D. Owing to the low amount of generated O—O species in 811‐D, the 1460 cm^−1^ signal is hardly detectable. Based on these data, the O‐oxidized species is closer to O_2_ in nature in 811‐A than in 811‐D. Therefore, it was concluded that the OAR in 811‐A was deeper than that in 811‐D. Further support will be provided in the following sections. For clearer distinction, the products of oxidized O in 811‐A and 811‐D are, respectively, called bulk‐O_2_‐like species and (O_2_)*^n^* ^−^.

The relative area of the elastic peak in the O–K‐edge RIXS spectra reflects the O‐hole content.^[^ ^36^ ^]^ Figure S22 (Supporting Information) shows the relative area evolution of the elastic peaks for the materials in different electrochemical states. As shown in Figure 3c, the elastic peak of 811‐A increases sharply in the 3.8 V charged electrode and declines in the 4.5 V charged electrode. In addition, the intensity of the elastic peak of the 3.0 V discharged 811‐A increases remarkably again. This interesting phenomenon is related to the incomplete reduction of the oxidized O species to O^−^ in 811‐A. For 811‐D, the elastic peaks are almost identical for the pristine and discharged electrodes, indicating improved reversibility for the O redox. The restrained change in the elastic peaks for the charged 811‐D electrodes verified the alleviated OAR in 811‐D. The elastic peaks and OAR fingerprint peaks indicate a related loss–gain relationship, which can be interpreted as indicating that O holes transform into other oxidized species at a deep charge. This finding is consistent with previously reported theoretical calculations of the lattice O redox path.^[^ ^37^ ^]^

Next, the soft‐synchrotron X‐ray absorption spectroscopy (XAS) spectra of the O–K‐edge in the total electron yield (TEY) and total fluorescence yield (TFY) modes for pristine and 4.5 V charged electrodes were tested. For the TEY spectra (Figure S23, Supporting Information), wider pre‐K‐edge peaks at ≈528 eV are observed for pristine 811‐A compared to pristine 811‐D, which indicates that 811‐A has a more complex electronic structure. In addition, when the electrodes were charged to 4.5 V, a more severe O redox reaction occurred in 811‐A than in 811‐D, as evidenced by the OAR signal at 532 eV. The TFY spectra in Figure 3d also indicate an obvious oxidized O signal in the 4.5 V charged 811‐A electrode. However, this signal in 811‐D is considerably restrained. These results demonstrate that the OAR in 811‐A is bulk‐sensitive, whereas 811‐D inhibits OAR.

Furthermore, the detailed electronic evolution of the lattice O on the surface of 811‐A and 811‐D was characterized by XPS. An interesting phenomenon is observed. Compared with the pristine state (Figure S24, Supporting Information), an obvious signal of bulk‐O_2_‐like species is distinctly spotted in the spectra of 3.7 V (Figure 3e), 4.1 V (Figure 3f), and 4.5 V (Figure 3g) charged 811‐A, but not in the other states of 811‐A (Figure S25, Supporting Information). No bulk‐O_2_‐like species signal is evident for the 811‐D electrodes. The fitting of the XPS plots for the etched electrodes, which eliminated the effect of surface adsorption, intuitively indicates this point (Figure S26, Supporting Information). The operando differential electrochemical mass spectrum (DEMS) detects O_2_ release at the three main voltages for 811‐A, corresponding to the phase transitions of H1→M, M→H2, and H2→H3 (Figure 3g), which is consistent with the XPS results.

The electronic structure of metals is closely related to their OAR. As shown in Figure S27 (Supporting Information), the bulk Ni evolution of the metals is reflected by the soft‐XAS results in the TFY mode. It should be noted that the spectra of the pristine electrodes are significantly different, which is related to the distinct Ni spin states of the materials. A high spin state causes 811‐A to exhibit high absorption at low energies.^[^ ^38^, ^39^ ^]^ The detailed evolution of the Ni XPS spectra in Figure S28 (Supporting Information) exhibits quicker Ni oxidation for 811‐A than 811‐D, as demonstrated by the earlier appearance of the Ni^4+^ signal in 811‐A. This finding demonstrates that with delithiating, an electron‐deficient state appears earlier for 811‐A than for 811‐D, which supports the faster emergence and deeper oxidation of the lattice O products for 811‐A.

### 2.4 Structural Evolution

**Figure 4:**
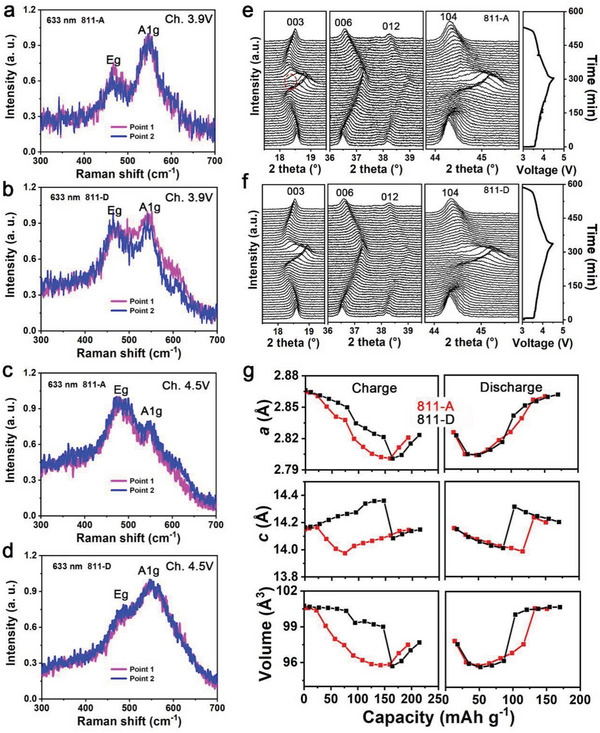
a,b) Raman spectra for 3.9 V charged 811‐A electrode and 811‐D electrode. c,d) Raman spectra for 4.5 V charged 811‐A electrode and 811‐D electrode. e,f) In situ XRD plots of 811‐A and 811‐D. g) Refined crystal parameters for two materials. Ni K‐edge. h,i) Wavelet transform of Ni K‐edge EXAFS spectra for 811‐A electrodes and 811‐D electrodes. Ch. stands for charge, and Dis. stands for discharge.

A Raman experiment with a 633 nm laser was conducted. To detect the structural properties accurately, multiple points on each sample were selected for testing. The shapes, shifts, and relative intensities of the O—Ni(Co/Mn)—O bending (*E*_g_) and Ni(Co/Mn)—O stretching (*A*_1g_) peaks reflect the structural features of Ni(Co/Mn)O_6_ octahedrons. Both materials exhibit broad peaks in the spectra of the pristine electrodes (Figure S29, Supporting Information). The peak intensity is slightly higher for *A*_1g_ than *E*_g_. However, the situation is complicated for charged electrodes. In the 3.9 V charged state (**Figure** **4**a,b), the *A*_1g_ peak of 811‐A is considerably intensified, reflecting the enhanced asymmetric bending vibration of O—Ni(Co/Mn)—O, which is derived from the unequal variation of cell parameters *a* and *b*.^[^ ^40^ ^]^ The 3.9 V charged 811‐D maintains a spectral shape similar to that of pristine 811‐D. When charging to 4.5 V, the elevated *E*_g_ peak in the 811‐A spectrum indicates that the O—O reverse vibration along the *c*‐axis is enhanced owing to the shrinkage in parameter *c*.^[^ ^40^ ^]^ This finding indicates that with delithiating, 811‐A experiences a faster structural change than 811‐D at depths of ≈100 nm for the particles.

The structural evolution of 811‐A and 811‐D upon (de)lithiation was subsequently probed by in situ XRD (Figure 4e,f). A fatigued phase is evident for delithiated 811‐A but not for delithiated 811‐D. The *a*, *c*, and volume (*V*) evolutions accompanied by (de)lithiation of 811‐A and 811‐D were revealed by Rietveld refinement (Figure 4g). Some special nodes for structural changes have been discovered. At ≈50 and 150 mAh g^−1^, the structural parameters of 811‐A underwent an abrupt change. For the structural parameters of 811‐D, only one quick change was observed, at ≈150 mAh g^−1^. These two capacities correspond to the phase transitions H1→M and M→H2. It should be noted that the rapid changes in the structural parameters caused by the H1→M phase transition reflect the obvious structural distortion of 811‐A at early charge, which is consistent with the above results. This special structure change of 811‐A was deduced to be generated by aggregative Li/Ni antisite defects. In addition, at discharge, these parameters show a delayed tendency to return to the pristine state for 811‐A compared with those for 811‐D. The corresponding XRD spectra at specific capacities were then compared (Figure S30, Supporting Information). Compared with 811‐D, 811‐A exhibits a faster peak migration in the charging process from 0 to 200 mAh g^−1^. Moreover, unlike the 104 peak in 811‐D, which maintains its form well, the 104 peak in 811‐A is considerably widened, showing a splitting tendency at ≈35 mAh g^−1^. Upon charging to ≈160 mAh g^−1^, splitting is detected in 811‐A. In addition, at ≈200 mAh g^−1^, a fatigued phase is evident in 811‐A, but not in 811‐D. After cycling, the XRD pattern of 811‐A shows obvious peak splitting, whereas this splitting is well restrained in 811‐D, indicating a difference in structural degradation between the two materials (Figure S31, Supporting Information). The cross‐sectional SEM images reveal cracks in the cycled electrode for 811‐A but not for 811‐D (Figure S32, Supporting Information).

**Figure 5:**
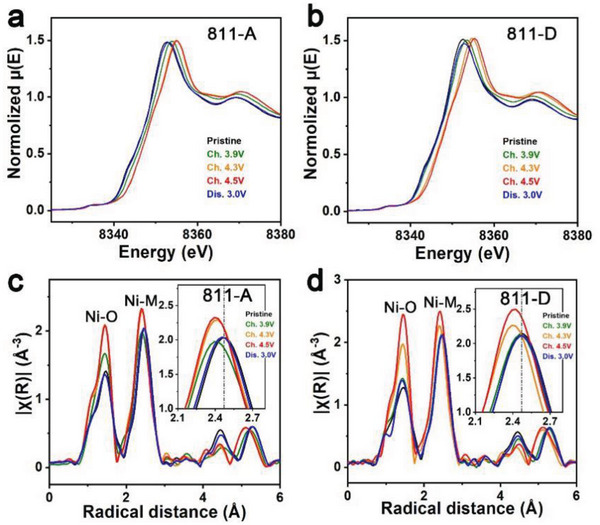
a,b) Ni K‐edge XAS spectra for 811‐A electrodes and 811‐D electrodes at different charge states. c,d) Fourier‐transformed Ni K‐edge EXAFS spectra of electrodes for 811‐A and 811‐D at different charge states. Ch. stands for charge, and Dis. stands for discharge.

The local environment of Ni was probed using XANES spectroscopy (**Figure** **5**a,b). A quicker absorption edge shift of Ni for 811‐A than for 811‐D from the pristine state to the 3.9 V charged state is evident. In addition, the spectra of 811‐A in the 4.3 V and 4.5 V charged states almost coincide. The Ni edge for 811‐D is still shifted toward a higher energy from the 4.3 V charged state to the 4.5 V charged state. Compared with 811‐D, 811‐A delivers inferior Ni redox reversibility, as indicated by the differences in the spectra of the pristine and 3.0 V discharged electrodes. The white‐line peak positions for these spectra intuitively reflect these changes (Figure S33, Supporting Information).

The k^2^‐weighted Fourier transform magnitudes from the XANES results for 811‐A and 811‐D are shown in Figure 5c,d, respectively. The first two peaks reflect the Ni—O and Ni—M (M: metal) shells. For 811‐A, the Ni—M shell obviously shrinks from the pristine state to the 3.9 V charged state. However, this change is not evident for the corresponding states of 811‐D. Moreover, the Ni—M shell shift in the spectra from the pristine state to the 4.5 V charged state is larger for 811‐A than for 811‐D. Compared with 811‐D, the structural distortion of 811‐A during charging appears earlier and larger, which is consistent with the above characterizations. The key differences between 811‐A and 811‐D upon delithiation appear near the nodes for the phase changes H1→M, M→H2, and H2→H3. A deep relationship among the OAR chemistry, structural evolution, and Li/Ni mixing is confirmed.

### 2.5 Mechanism for Different O Redox Behaviors and Heterogeneities

**Figure 6:**
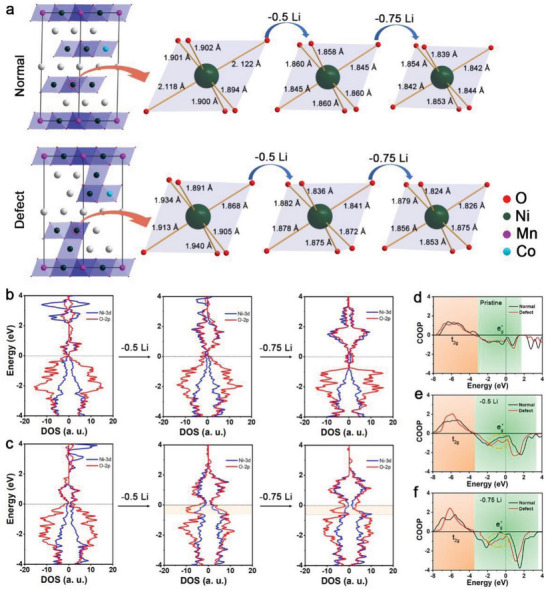
a) Calculated Ni—O bond lengths for a NiO_6_ octahedron in normal and defected Ni‐rich oxide models. b,c) Calculated DOS for Ni/O in the models for 811‐D and 811‐A. d–f) COOP analysis of Ni—O in pristine, 50% delithiated, and 75% delithiated states.

First‐principles calculations were performed to investigate the mechanism of Li/Ni antisite defects in OAR chemistry. For feasible computation, a normal Li_12_Ni_10_Mn_1_Co_1_O_24_ model was used to represent 811‐D, and a disordered (Li_10_Ni_2_)(Li_2_Ni_8_Mn_1_Co_1_)O_24_ model with a periodic Li/Ni disorder‐Li/Ni order‐Li/Ni disorder configuration was used to represent 811‐A. The NiO_6_ octahedral transformations in Li_12_Ni_10_Mn_1_Co_1_O_24_ and (Li_10_Ni_2_)(Li_2_Ni_8_Mn_1_Co_1_)O_24_ with delithiation were calculated (**Figure** **6**a). The Ni—O bond length distribution in (Li_10_Ni_2_)(Li_2_Ni_8_Mn_1_Co_1_)O_24_ was more discrete than that in Li_12_Ni_10_Mn_1_Co_1_O_24_, which signified greater structural distortion of (Li_10_Ni_2_)(Li_2_Ni_8_Mn_1_Co_1_)O_24_. This result is consistent with the experimental results. Simultaneously, the evolution of the electronic structure of Li_12_Ni_10_Mn_1_Co_1_O_24_ and (Li_10_Ni_2_)(Li_2_Ni_8_Mn_1_Co_1_)O_24_ with different amounts of intercalated Li was probed through the density of states (DOS).

As shown in Figure 6b, the spin‐up and spin‐down states of Ni and O in Li_12_Ni_10_Mn_1_Co_1_O_24_ are almost symmetric, which is related to the electron configuration. In total, 90% of Ni cations in Li_12_Ni_10_Mn_1_Co_1_O_24_ appear as Ni^3+^ with an electron configuration of *t*_2g_^6^ *e*_g_^1^ in the pristine state. Upon delithiation, they are gradually oxidized to Ni^4+^ with an electron configuration of *t*_2g_^6^ *e*_g_^0^. Under these conditions, the numbers of up‐spin and down‐spin electrons are largely equal, resulting in similar up‐spin and down‐spin states. The up‐spin and down‐spin states of Ni in pristine (Li_10_Ni_2_)(Li_2_Ni_8_Mn_1_Co_1_)O_24_ are evidently different (Figure 6c). The calculated DOS plots of Ni/O indicate a larger electron‐occupied state span for 811‐A than for 811‐D, which verifies the enlarged energy band spans of 811‐A (Figure S34, Supporting Information).

This asymmetry becomes increasingly evident with delithiation. After removing 50% or 75% Li from (Li_10_Ni_2_)(Li_2_Ni_8_Mn_1_Co_1_)O_24_, a signal of enhanced electron density appears near the Fermi level on the DOS of O, which enhances the electrochemical activity of O. This behavior does not occur for Li_12_Ni_10_Mn_1_Co_1_O_24_. Combined with the crystal orbital overlap population (COOP), part of the *e*_g_^*^ state near the Fermi level in delithiated (Li_10_Ni_2_)(Li_2_Ni_8_Mn_1_Co_1_)O_24_ is more intense than that in delithiated Li_12_Ni_10_Mn_1_Co_1_O_24_ (Figure 6d–f). The increase in antibonding is believed to be correlated with the enhanced O electron density near the Fermi level, which increases the instability of the structure.

Structural distortion influences the electronic structure of materials by affecting crystal field band splitting. As illustrated in Figure S35 (Supporting Information), in a regular octahedron, the five degenerate 3*d* orbitals of Ni split into triple‐degenerate *t*_2g_ orbitals and double‐degenerate *e*_g_ orbitals under the action of the crystal field, which evolve to *e*_g_* and *t*_2g_ bands after orbital hybridization. In the deformed octahedron, due to the complex interactions of ligand O on the central Ni, *t*_2g_ and *e*_g_ orbitals with reduced degeneracy are produced, and thus *e*_g_* and *t*_2g_ bands with elongated energy spans evolve. Ni in the deformed octahedron exhibits more single electrons than that in the regular octahedron because of the increase in the spin state, which explains the difference in symmetry between the up‐spin and down‐spin states of Li_12_Ni_10_Mn_1_Co_1_O_24_ and (Li_10_Ni_2_)(Li_2_Ni_8_Mn_1_Co_1_)O_24_.

**Figure 7:**
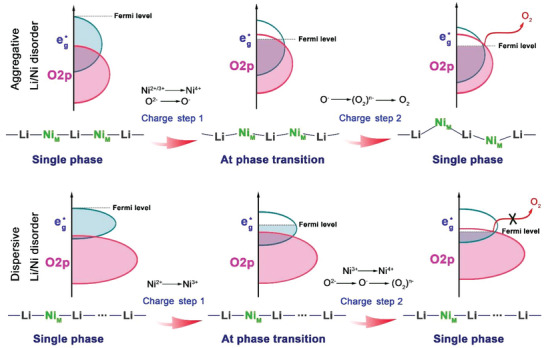
Sketches illustrating the interactions of Li/Ni antisite defect/OAR chemistry/heterogeneity. Ni_M_ means the migrated Ni in the Li layer.

Based on the above results and discussions, a full understanding of the interactions of the Li/Ni antisite defect/OAR chemistry/chemo‐mechanical heterogeneity was obtained, as shown in **Figure** **7**. Overall, the difference in the band structure is the cause of the different charge‐compensation mechanisms of the two materials. The spin state of Ni plays two functions in influencing the redox mechanisms. First, the Ni spin state affects the redox mechanism by influencing the band structure. Here, 811‐A exhibits aggregative Li/Ni mixing, and 811‐D exhibits dispersive Li/Ni mixing. Compared with dispersed Li/Ni mixing, aggregative Li/Ni mixing increases the spin state of Ni. Because adjustment of the Ni spin state from low to high complicates the electronic structure, the band spans are elongated. The electronic state at the Fermi level is enhanced for 811‐A because of its high Ni spin state. In the oxidation process, both samples begin reactions with Ni oxidation, which corresponds to electron stripping from the *e*_g_^*^ band. Owing to the electronic state enhancement at the Fermi level, 811‐A shows deeper Ni oxidation than 811‐D at relatively low charge voltages. Simultaneously, because 811‐A has a longer O2p band span than 811‐D, the Fermi level approaches the O2p band at a lower charge voltage for 811‐A than for 811‐D. Thus, the triggered O2p→Ni *e*_g_^*^ charge transfer and resulting O redox reaction appear earlier for 811‐A than for 811‐D. Second, the spin state of Ni influences the structural distortion. Because Ni in the high‐spin state increases the complexity of the structure and charge compensation of 811‐A, a more obvious distortion for 811‐A than for 811‐D is demonstrated with delithiating. In particular, in the phase transitions H1→M, M→H2, and H2→H3, when the lattice displacement is easily formed, the structural distortion is intensified. This phenomenon could causes the O2p band to exceed the Fermi level, leading to sharp O→Ni charge transfer and O_2_ release and resulting in a structural‐phase‐transition‐dependent OAR. O_2_ release and abundant bulk‐O_2_‐like species are observed in the H1→M, M→H2, and H2→H3 phase transitions in 811‐A. The O_2_ release and structural degradation finally account for the severe chemomechanical heterogeneity of the cathode. This result supports that of a previous study that indicated that lattice displacement is the cause of the lattice O reaction.^[^ ^41^ ^]^ Dispersive Li/Ni disorder does not significantly influence the NiO_6_ octahedron, which restrains the OAR during phase transitions by reducing the intertwining between the O2p and *e*_g_* states.

The form of Li/Ni disorder (dispersive or aggregative) and degree of Li/Ni disorder are difficult to discuss separately. When the Li/Ni disorder degree is high, the Li/Ni mixing defects appear in an aggregated state, whereas when the Li/Ni disorder degree is low, the Li/Ni mixing defects appear in a dispersed state. Owing to the significant effect on the structure, electronic state changes caused by the aggregative Li/Ni disorder appear, inducing the production of high‐spin‐state Ni and ultimately promoting the OAR during charging and discharging. However, the electronic state changes caused by dispersive Li/Ni disorder are diluted by the surrounding chemical environment and thus cannot be fully manifested. Therefore, dispersive Li/Ni disorder does not promote the OAR.

## 3 Conclusion

In this work, we revealed a correlation between the OAR chemistry and Li/Ni antisite defects in Ni‐rich oxide cathodes for the first time. Distinct OAR chemistry was demonstrated using a cathode with aggregative or dispersive Li/Ni antisite defects. The characteristics of Li/Ni antisite defects play a decisive role in influencing the OAR chemistry by varying the Ni spin states and energy band spans. Specifically, 811‐A with aggregative Li/Ni antisite defects and an elevated Ni spin state exhibited an enlarged energy band span. Upon delithiation, severe NiO_6_ distortion and obvious competitive electron stripping between the O2p and *e*_g_^*^ bands occurred at nodes for the H1→M, M→H2, and H2→H3 phase changes. This behavior contributed to the abundant generation of bulk‐O_2_‐like species and O_2_ gas release in the corresponding states, which led to severe heterogeneity. In contrast, 811‐D exhibited a restrained OAR with dispersive Li/Ni antisite defects and a relatively low Ni spin state. O oxidation was restricted by the production of a small amount of (O_2_)*^n^* ^−^. After discharge, (O_2_)*^n^* ^−^ was completely reduced to lattice O^2−^, whereas the bulk‐O_2_‐like species were only partly reduced to O^−^. Thus, OAR reversibility and heterogeneity were fundamentally improved. Enhanced electrochemical kinetics, a high capacity at 0.1 C (>200 mAh g^−1^), and a good capacity retention at 0.2 C (93.8%@100 cycles) were delivered by 811‐D. This study revealed the critical functional mechanisms of different Li/Ni antisite defect characteristics on the OAR chemistry and chemo‐mechanical heterogeneity of LiNi_0.8_Co_0.1_Mn_0.1_O_2_. The insights regarding the importance of modulating Li/Ni antisite defects in tuning the OAR chemistry and restraining heterogeneity are applicable to other Ni‐rich layered cathodes as well.

## 4 Experimental Section

### Synthesis

To prepare the precursor for 811‐D, coprecipitation with a single precipitation time was adopted. First, 0.08 mol H_2_C_2_O_4_·2H_2_O (99.5%, MACKLIN) was dissolved in 80 mL distilled water to serve as the precipitating agent solution. It was added dropwise into an aqueous solution (50 mL) containing 0.08 mol NiSO_4_·6H_2_O (99%, MACKLIN), 0.01 mol CoSO_4_·7H_2_O (99%, MACKLIN), and 0.01 mol MnSO_4_·5H_2_O (99%, MACKLIN) with stirring. A green precipitate was obtained and washed with distilled water three times and with ethanol twice. Drying the green powder yielded the 811‐D precursor.

To produce the precursor for 811‐A, coprecipitation with two precipitations was adopted. The first precipitation event was the same as that described above. After the formation of the green precipitate, 0.001 NiSO_4_·6H_2_O and 0.001 H_2_C_2_O_4_·2H_2_O were re‐fed into the solution for the second precipitation. Washing and drying the green powder yielded the 811‐D precursor.

To form the 811‐D and 811‐A, the precursors of 811‐D and 811‐A were each uniformly mixed with 5% beyond the stoichiometric amount of LiOH·H_2_O (99%, MACKLIN). The mixed powders were calcined at 900 °C for 12 h in air.

### Characterization

The XRD data were collected using an X‐ray diffractometer (Cu K𝛼, 𝜆 = 1.5406 Å, Rigaku Smart Lab). The neutron powder diffraction experiments were conducted on an MPI from China Spallation Neutron Source (CSNS).^[^ ^42^ ^]^ The morphology of the samples was observed with a scanning electron microscope (Hitachi SU8010). The valence states of the elements in the samples were probed by X‐ray photoelectron spectroscopy (Al K*α* X‐ray source, AXIS Supra). A HORIBA LabRAM Odyssey was used to collect Raman spectra with wavelengths of 532 and 633 nm. An aberration‐corrected scanning transmission electron microscope JEM ARM200F (JEOL, Tokyo, Japan) equipped with two CEOS (CEOS, Heidelberg, Germany) probe aberration correctors was used to probe the structure at the atomic scale. The X‐band electron spin resonance spectra were obtained by a Bruker E500 spectrometer at 4 K. Time‐of‐flight secondary ion mass spectrometry analysis was conducted on a PHI nanoTOF II Time‐of‐Flight SIMS with a 30 keV Bi analysis ion beam. The chemical compositions of the materials were measured by an inductively coupled plasma optical emission spectrometer (Agilent 730). DEMS was conducted using a cell with gas inlet and outlet ports. Ar carrier gas was flown at a constant rate (0.2 mL min^−1^) through the cell and into a quadrupole mass spectrometer (Hiden HPR‐20).

### Electrochemical Measurements

The electrochemical performance of the samples was tested using CR2025 coin cells. First, 80 wt% active material, 10 wt%), super P carbon (10 wt%), and polyvinylidene fluoride were mixed evenly with N‐methyl pyrrolidinone. The slurry was deposited on Al foil and dried under a vacuum at 110 °C for 12 h. The typical loading of the active material was 2.5–3.0 mg cm^−2^. The electrolyte consisted of 1.15 m LiPF_6_ in ethylene carbonate, dimethyl carbonate, and ethyl methyl carbonate (1:2:2 vol%). A Celgard 2400 was used as the separator. The cells (CR2025) were assembled in an Ar‐filled glove box (O_2_, H_2_O < 0.1 ppm). Galvanostatic charging and discharging tests were performed using the NEWARE system. The cells were tested in an electrochemical window of 3.0–4.5 V (vs Li/Li^+^) unless otherwise stated. EIS and GITT were performed using an electrochemical workstation (Auto Lab). The 1 C capacity was defined as 200 mA g^−1^.

### Synchrotron Radiation Spectroscopy

The RIXS data were collected at the PEAXIS beamline of the synchrotron BESSY II at Helmholtz‐Zentrum Berlin.^[^ ^43^ ^]^ The hard XAS experiments were performed at beamlines 4B9A and 1W1B of the Beijing Synchrotron Radiation Facility. A double‐crystal monochromator was used in this study. The metal foil was measured before the XAS measurements to ensure that the data were calibrated in the case of any drift in the monochromator position. The electrodes were measured in transmission mode. Soft XAS was conducted at beamline 02B02 of Shanghai Synchrotron Radiation Facility (SSRF).

### First‐Principles Calculations

The density functional theory (DFT) calculations were performed using the Vienna Ab initio simulation package. The exchange and correlation functions were described by the generalized gradient approximation function with Perdew–Burke–Ernzerhof. A resolution of 2*π* × 0.04 Å^−1^ was adopted for the Uniform G‐centered k‐point meshes and Methfessel–Paxton electronic smearing. A cut‐off energy of 500 eV was used for the simulation. Convergence of the total energy to within 1 meV per atom was ensured. Structural relaxation continued until the atomic force was less than 1 meV Å^−1^, and the total stress tensor was within 0.01 GPa of the target value. The Coulomb interactions of the on‐site were described by calculating all the elementary reaction steps using the DFT+*U* approach, and the *U* terms of Mn‐3d, Ni‐3d, and Co‐3d were 5.0, 7.0, and 5.5 eV, respectively. The crystal orbital overlap population was calculated using the LOBSTER package with the same parameters used in the DOS calculations.

### Statistical Analysis

The Li/Ni disorder degrees for the surfaces of 811‐A and 811‐D were obtained from the peak area ratio of the line intensity in the Li layer/line intensity in the TM layer. The line intensities for the Li and TM layers were obtained from randomly selected areas of the Li and TM layers in the aberration‐corrected scanning transmission electron microscopy images using Digital Micrograph software.

## Conflict of Interest

The authors declare no conflict of interest.

## Supporting information

Supporting Information

## Data Availability

The data that support the findings of this study are available from the corresponding author upon reasonable request.
