# Supplementary material for: Oxygen Anion Redox Chemistry Correlated with Spin State in Ni‐Rich Layered Cathodes
Source: Adv Sci (Weinh). 2023 Jan 25;10(9):2206442. doi: 10.1002/advs.202206442 (PMC10037688; doi:10.1002/advs.202206442)
Supplement: Supplementary file 1 — Supporting Information [file ADVS-10-2206442-s001.pdf]

Supporting Information

Oxygen Anion Redox Chemistry Correlated with Spin State in Ni-rich Layered Cathodes

Zhihua Lu,[∥1] *Jicheng Zhang,*[∥1]* *Qinghua Zhang,*[2] *Deniz Wong,*[3] *Wen Yin,*[4] *Nian Zhang,*[5] *Zhongjun Chen,*[6] *Lin Gu,*[2] *Zhongbo Hu,*[1] *and Xiangfeng Liu*[1,7]*

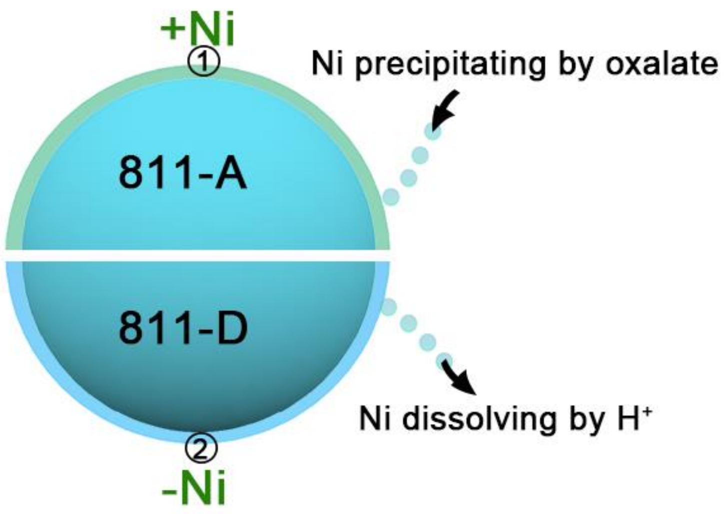

Figure S1. Schematic synthesis of the precursors for 811-A and 811-D.

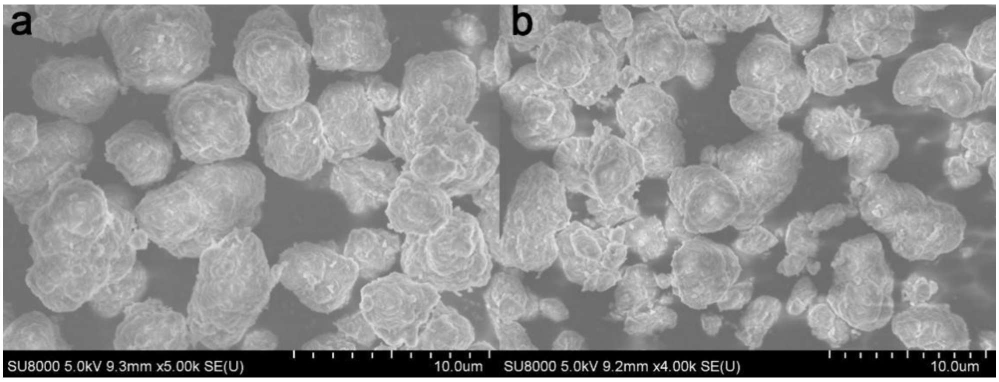

Figure S2. SEM images of precursors for 811-D (a) and 811-A (b).

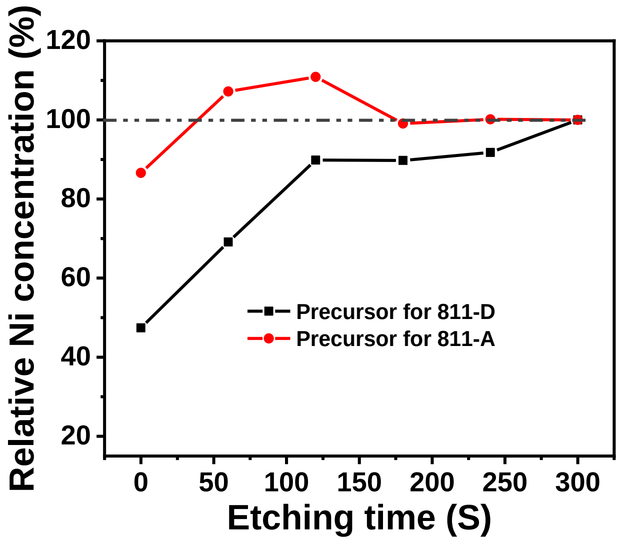

Figure S3. The detected relative Ni concentration for precursors of 811-D and 811-A through XPS.

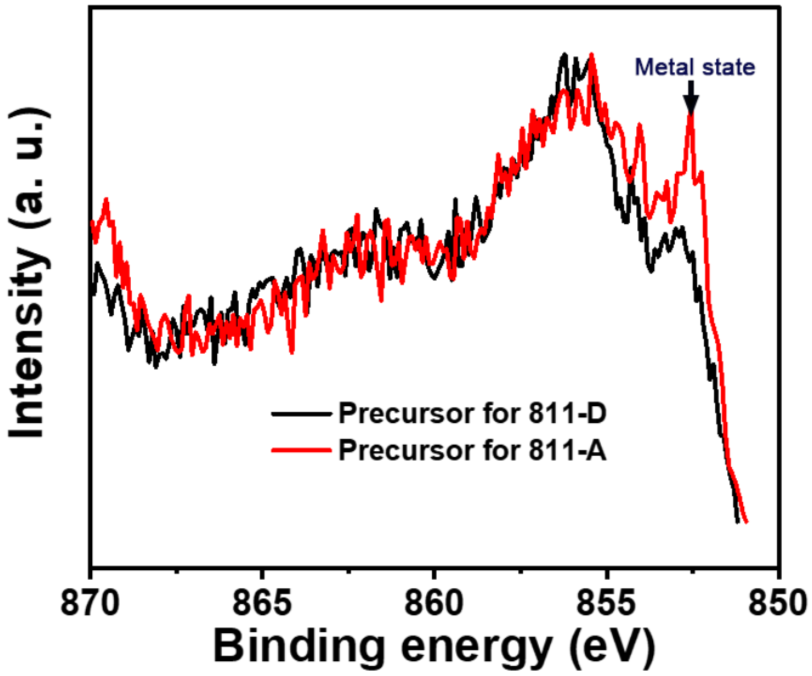

Figure S4. The Ni 2p XPS spectro for the precursors with a 150 °C heating treatment.

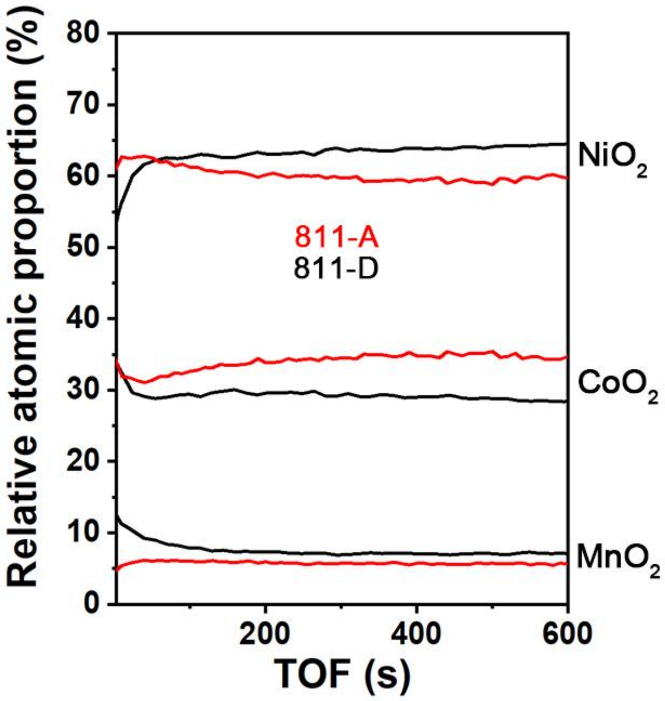

Figure S5. TOF-SIMS depth profiles of NiO_2, CoO_2, MnO_2.

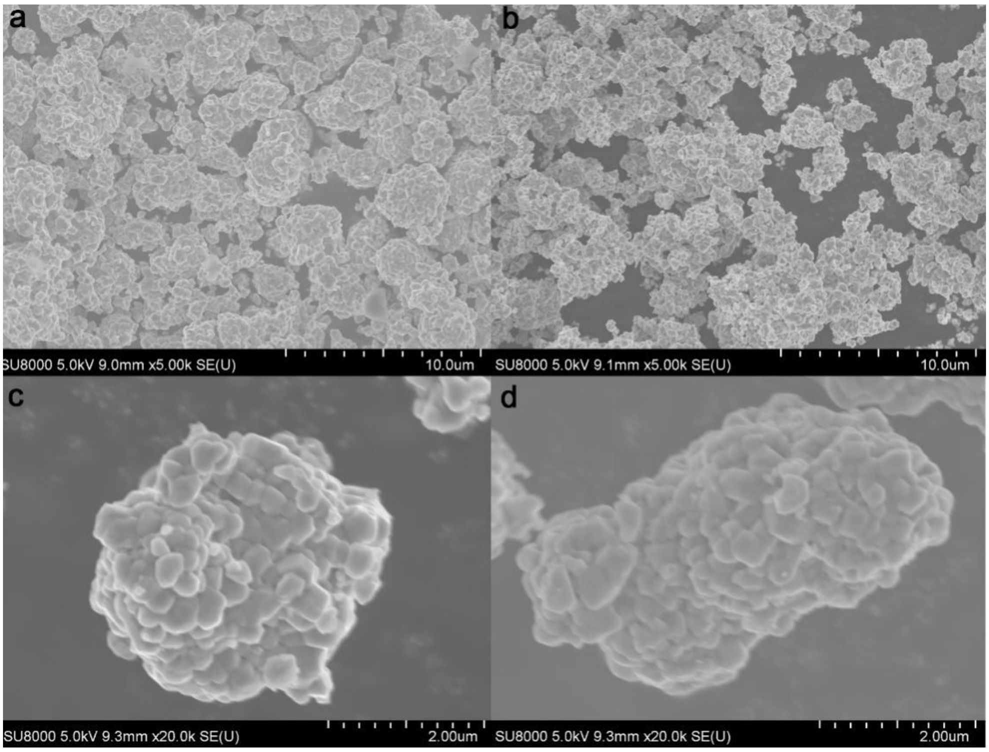

Figure S6. SEM images of 811-D (a) and 811-A (b) at a low magnification. SEM images of 811-D (c) and 811-A (d) at a high magnification.

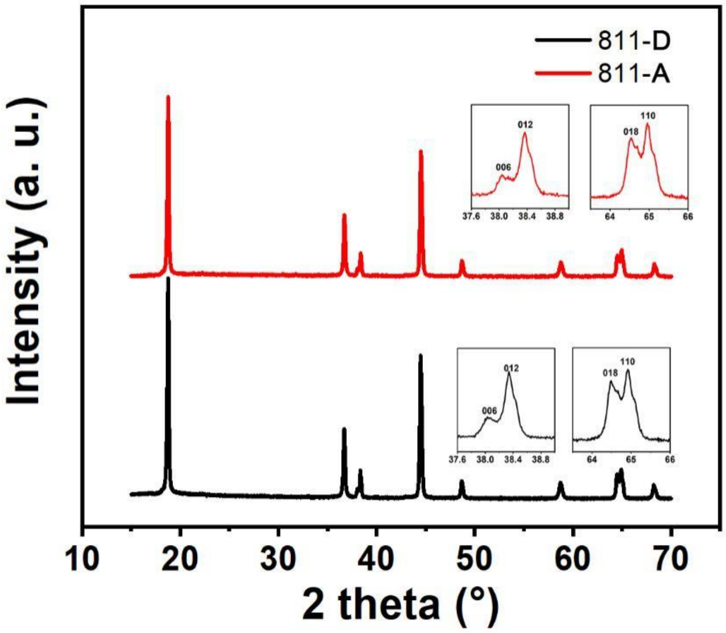

Figure S7. XRD plots of 811-A and 811-D.

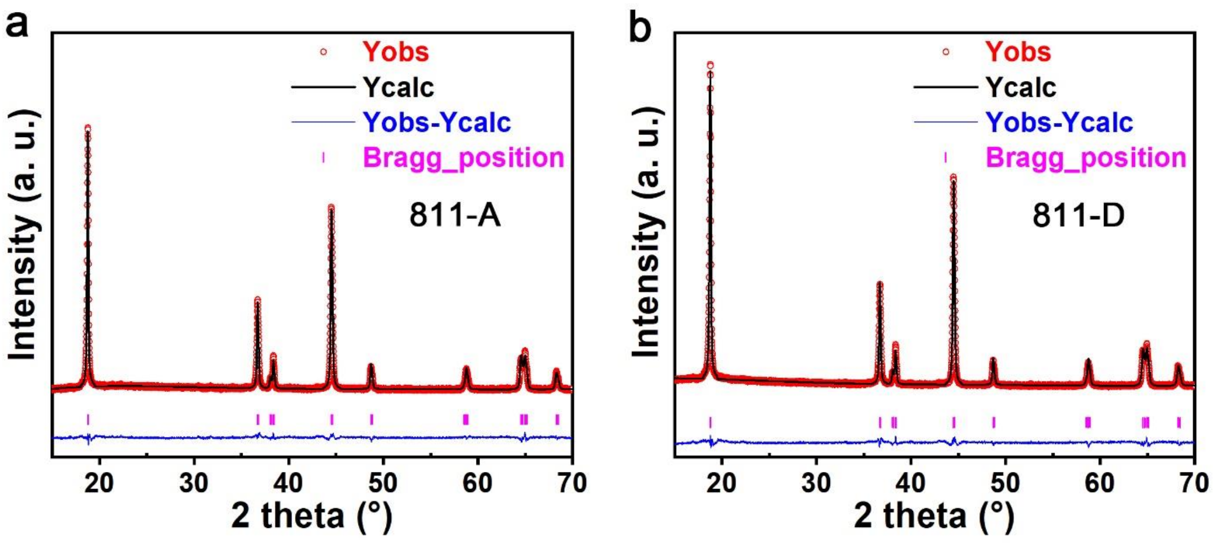

Figure S8. XRD Rietveld refinements for 811-A (a) and 811-D (b).

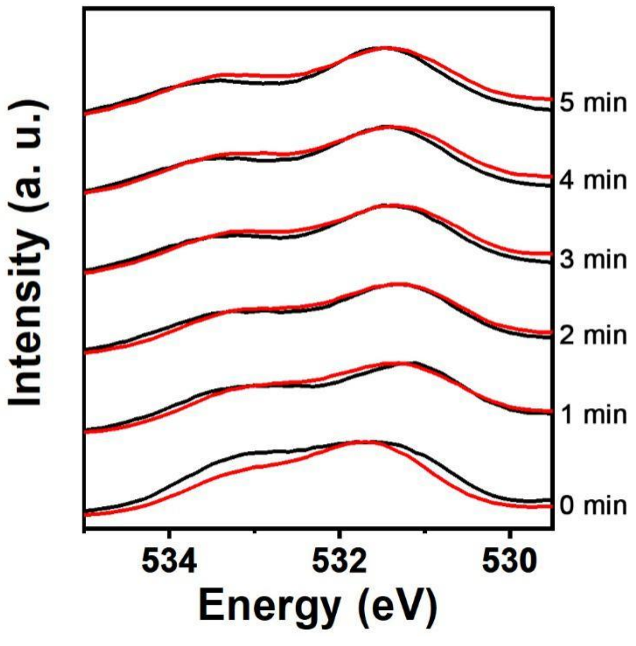

Figure S9. O2p XPS spectra for 811-A (red) and 811-D (black) at different etching time with Ar^+.

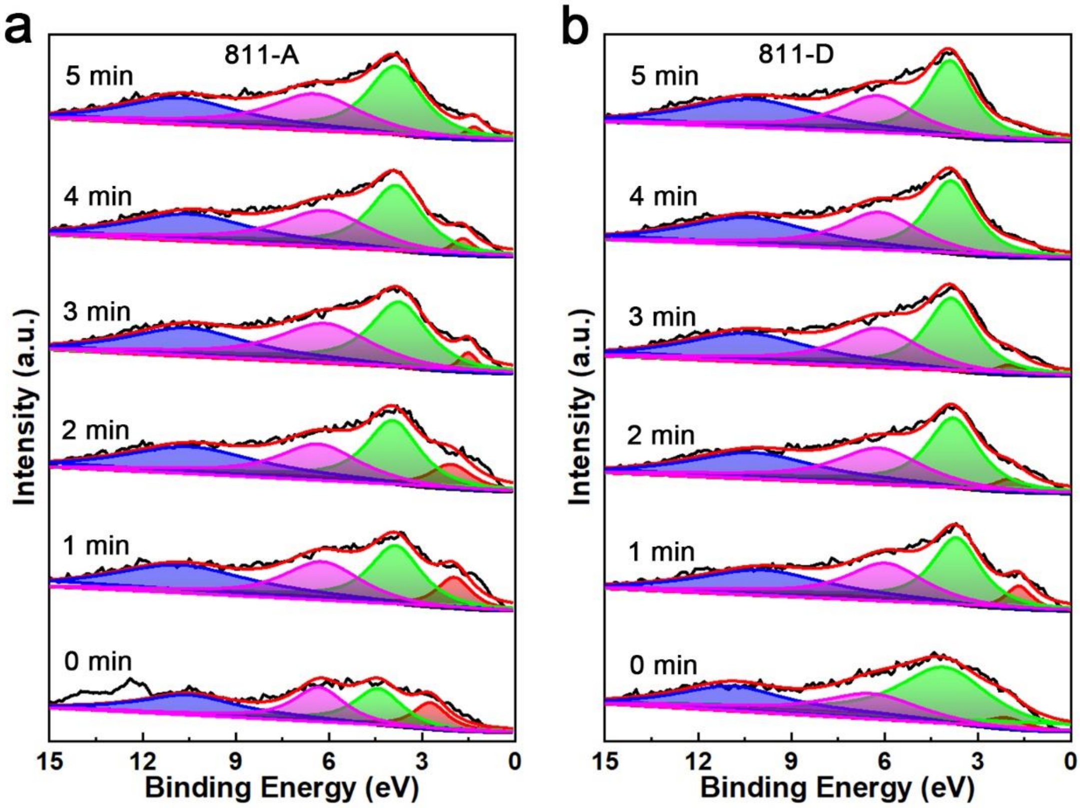

Figure S10. VB XPS spectra at different etching time with Ar^+ for 811-A (a) and 811-D (b).

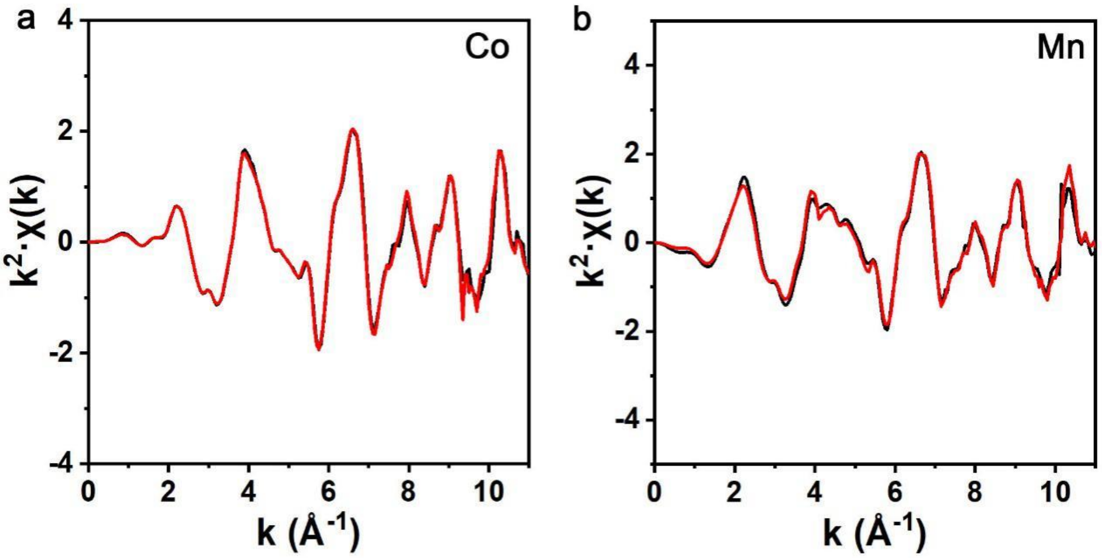

Figure S11. The raw Co (a) and Mn (b) EXAFS data. Red represents 811-A, black represents 811-D.

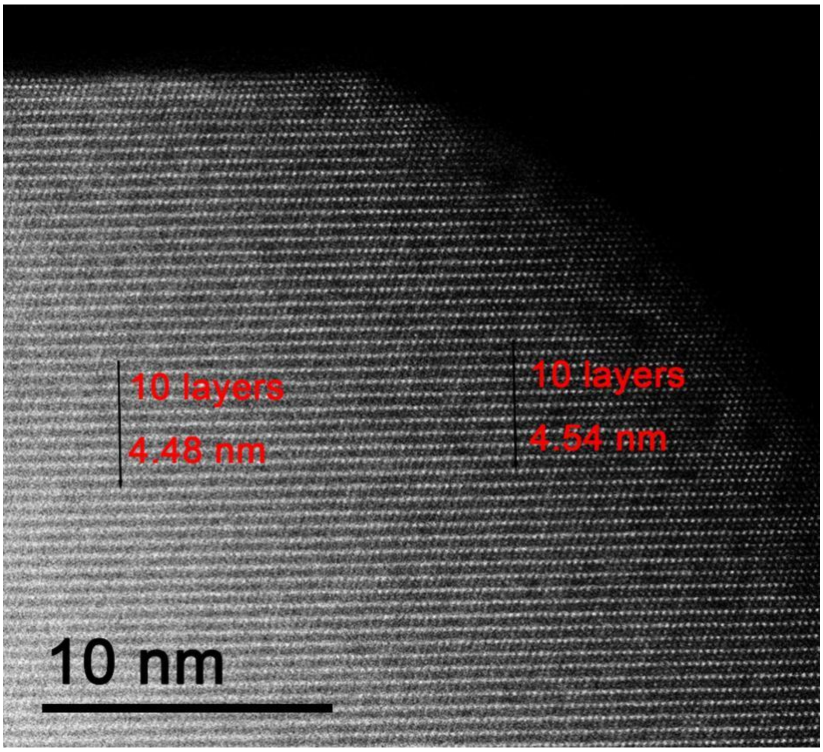

Figure S12. AC-STEM image for 811-A.

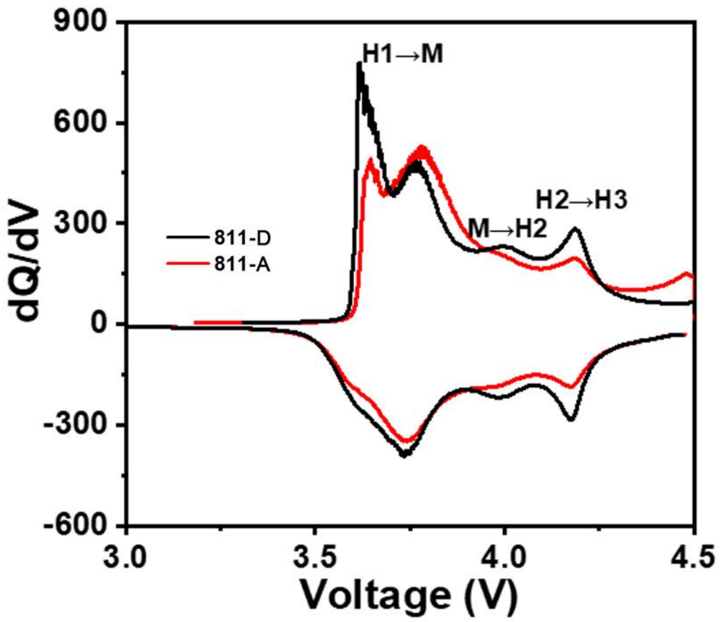

Figure S13. *d*Q/*d*V plots of the initial charge-discharge curves for 811-A and 811-D.

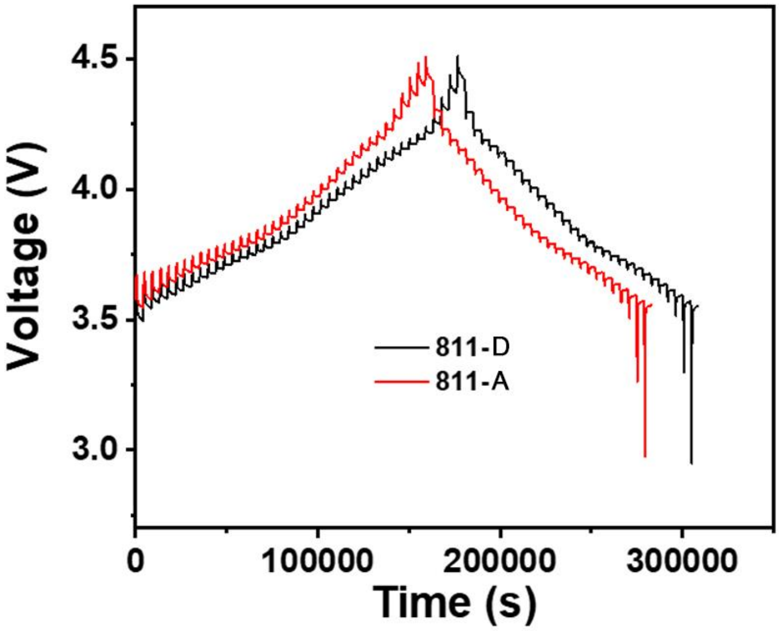

Figure S14. The galvanostatic intermittent titration (GITT) plots. Li^+ diffusion coefficient is calculated through the equation: $D_{Li^+} = \frac{4}{\pi\tau}\left(\frac{m_B V_m}{M_B S}\right)^2\left(\frac{\Delta E_s}{\Delta E_\tau}\right)^2$, where τ, m_B, V_m, M_B and S respectively stand for the time duration during the current pulse, active material mass on the electrode, molar volume of active material, molecular weight of active material, and the contact area between the electrolyte and the electrode. ΔE_s is the difference in the steady-state voltage at a single-step GITT experiment, and ΔE_τ is the potential change during charging or discharging at the time of current of flux after subtracting the IR drop.

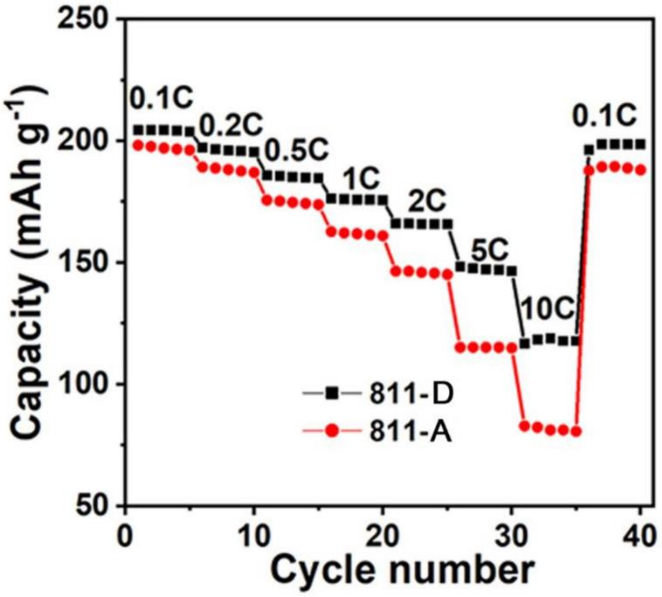

Figure S15. The rate performances of 811-A and 811-D.

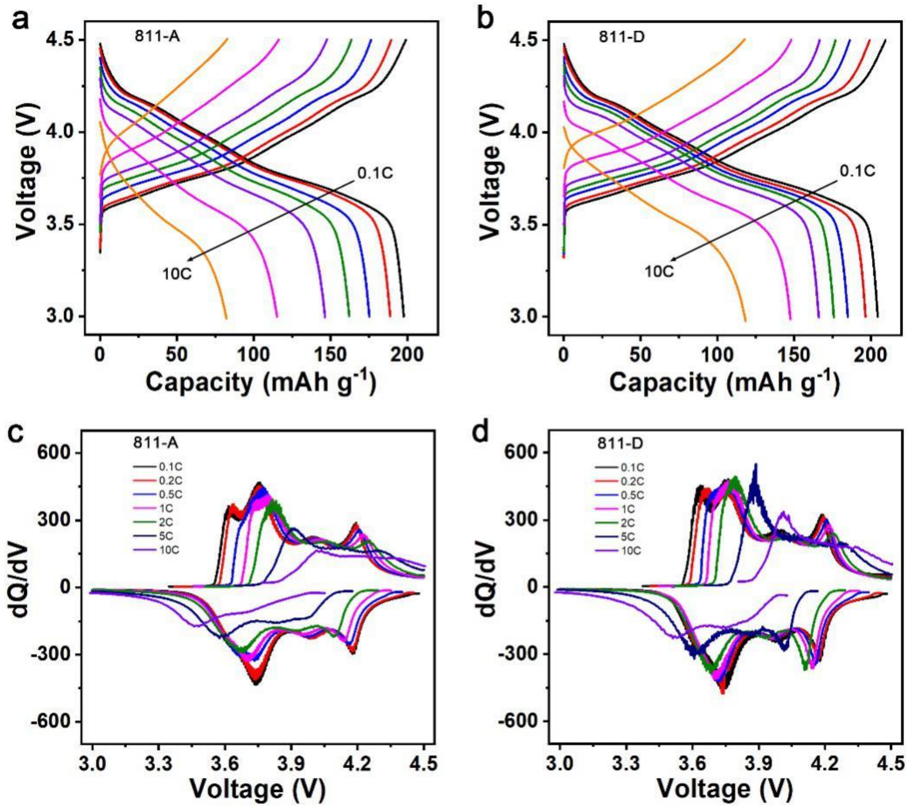

Figure S16. The charge-discharge curves of 811-A (a) and 811-D (b) at different rates. The *d*Q/*d*V plots of 811-A (c) and 811-D (d) at different rates.

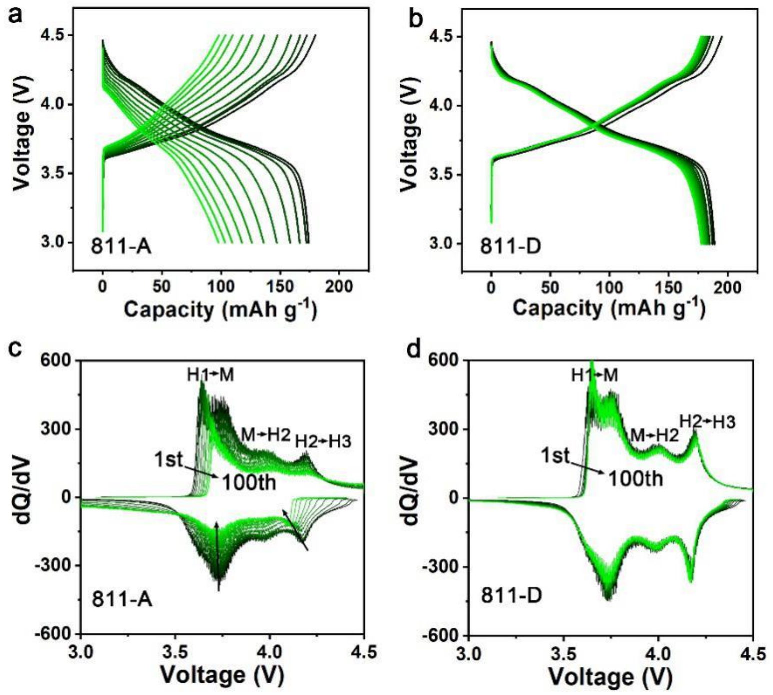

Figure S17. The charge-discharge curves of 811-A (a) and 811-D (b) from 1th to 100th cycle at 0.2 C. The dQ/dV plots of 811-A (c) and 811-D (d) from 1th to 100th cycle at 0.2 C.

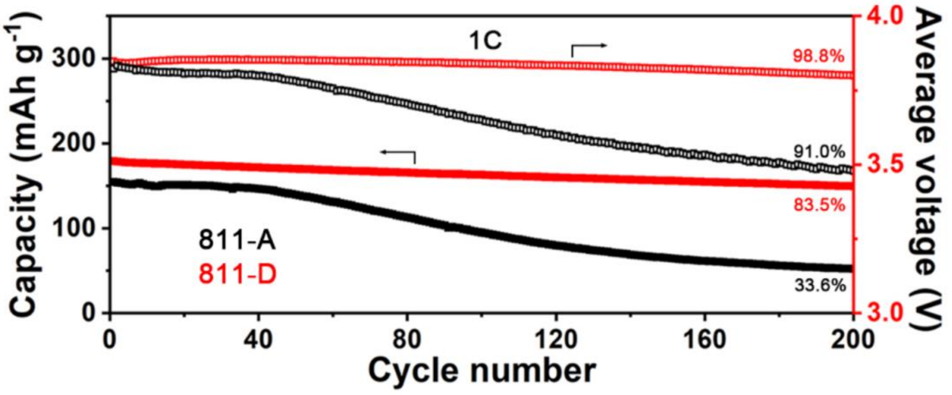

Figure S18. The cycling performances of 811-A and 811-D at 1 C.

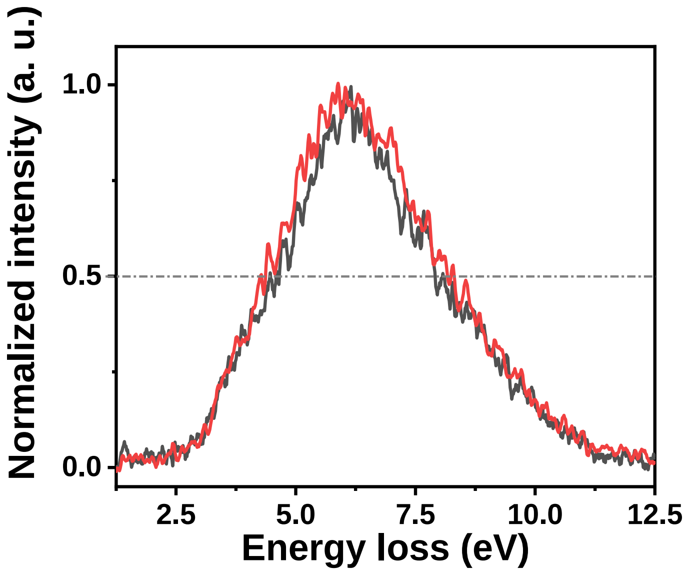

Figure S19. The comparison for normalized O-K edge RIXS spectra for electrodes at pristine state. Red represents 811-A, black represents 811-D.

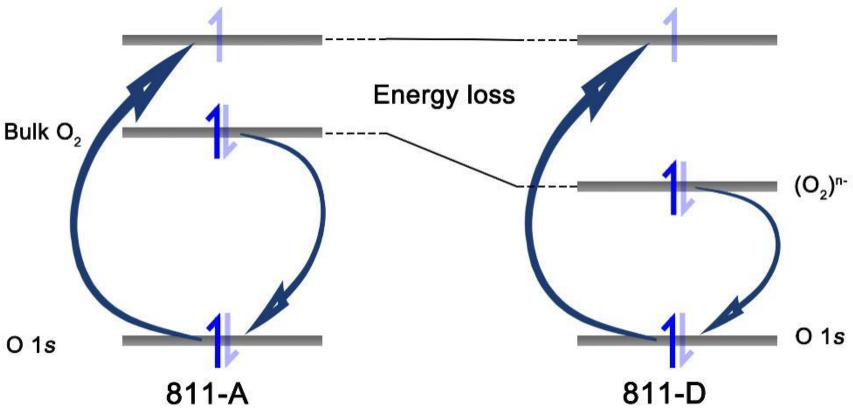

Figure S20. RIXS process for oxidized O feature peak in 811-A and 811-D.

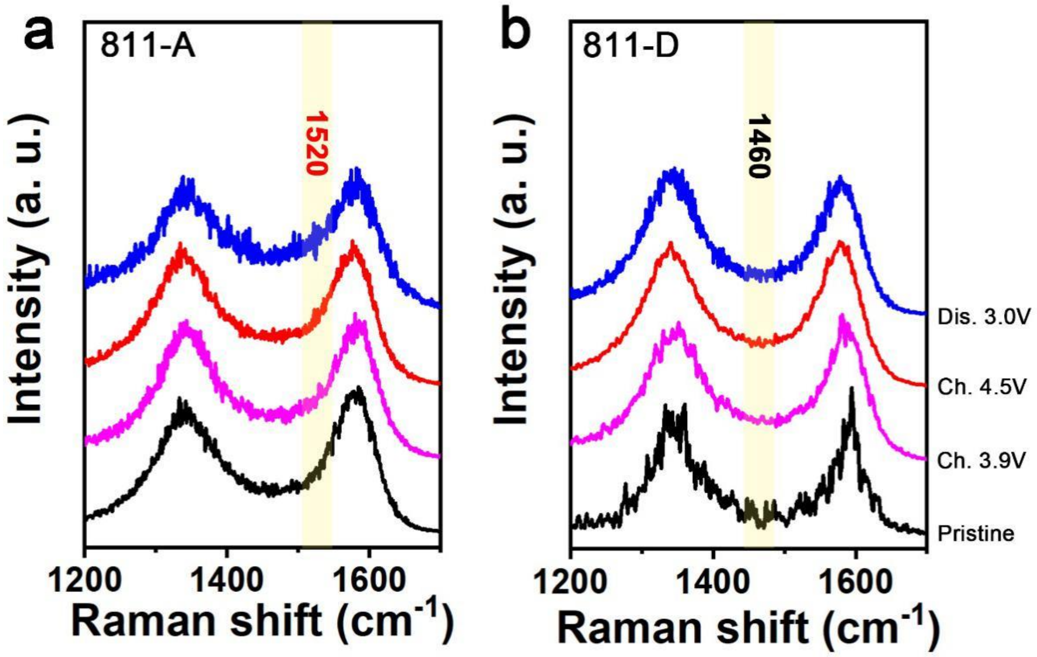

Figure S21. Raman spectra for 811-A (a) and 811-D (b) at different states of charge.

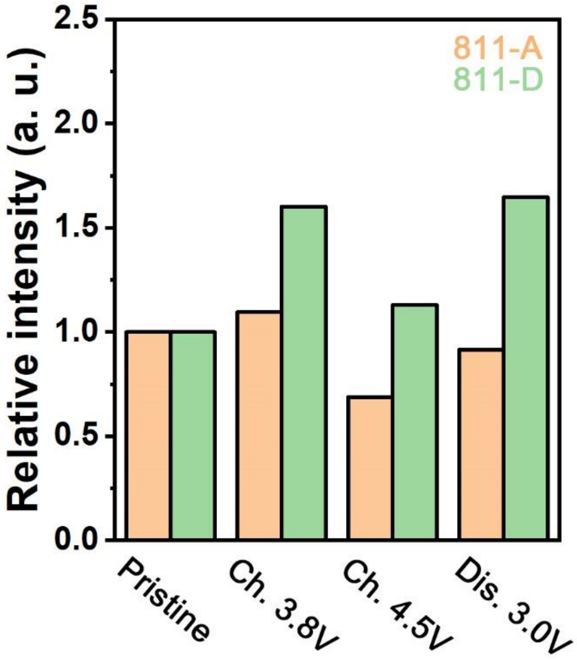

Figure S22. The relative areas of the elastic peaks for 811-A and 811-D at different states of charge. Setting the elastic peak area of each sample in the pristine state as 1.

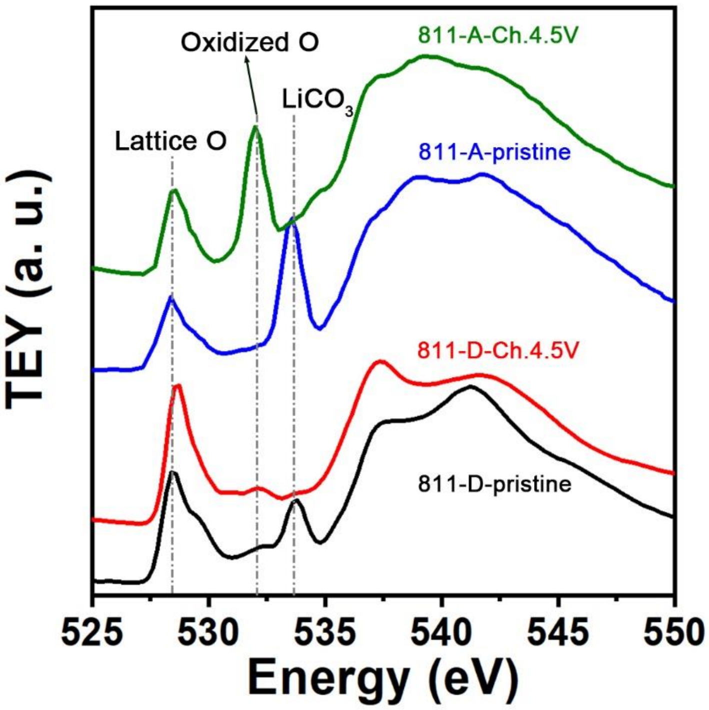

Figure S23. The O–K-edge spectra for pristine and 4.5V charged electrodes in TEY modes.

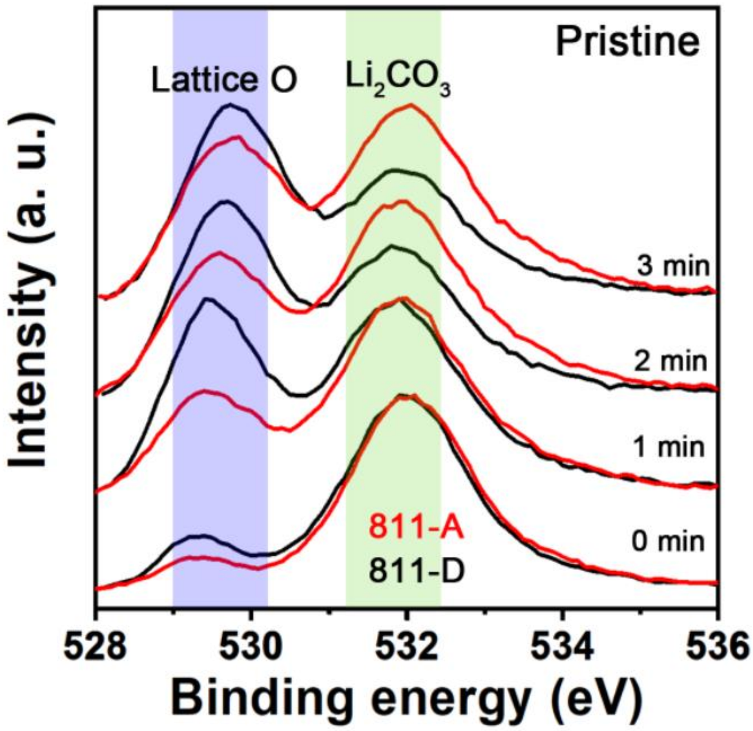

Figure S24. The O 2*p* XPS depth spectra for pristine electrode.

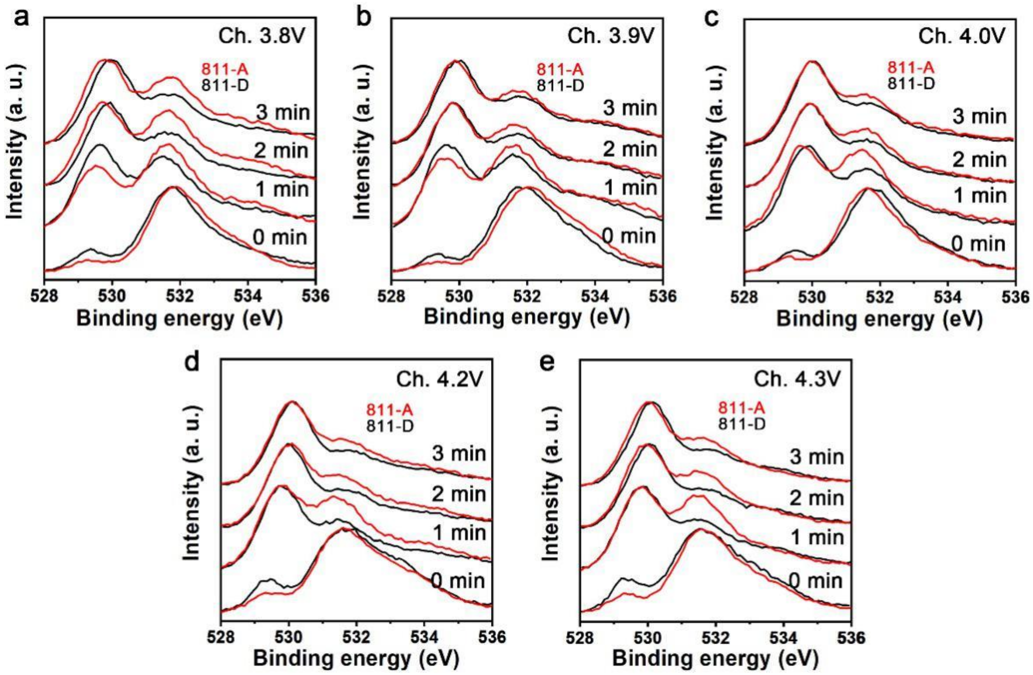

Figure S25. The O 2*p* XPS depth spectra for 3.8V-charged (a), 3.9V-charged (b), 4.0V-charged (c), 4.2V-charged (d), 4.3V-charged (e) electrodes.

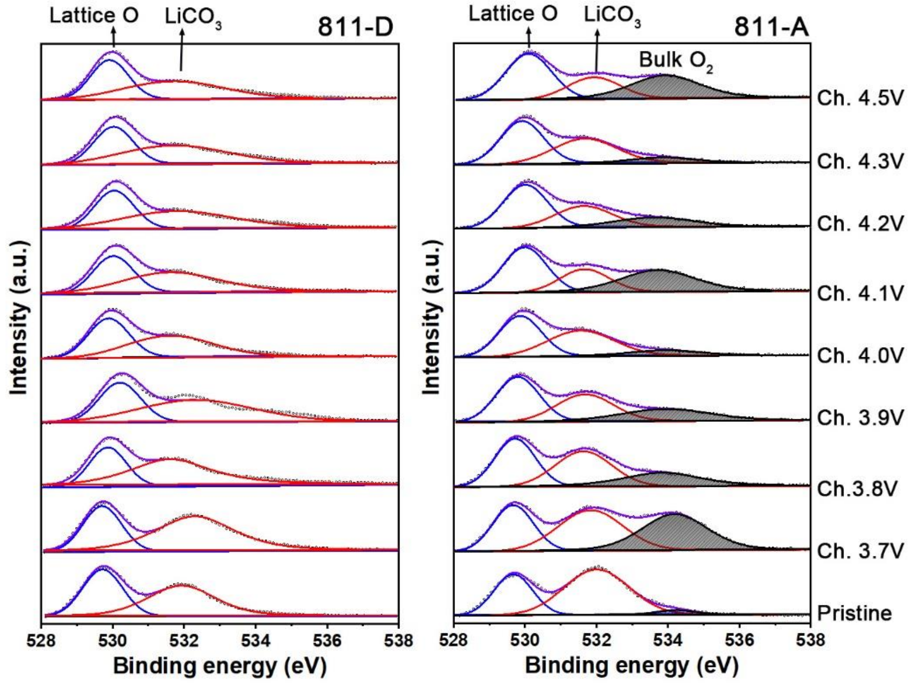

Figure S26. The fitting of the O1s XPS plots for 811-D and 811-A electrodes with Ar^+ etching for 3 minutes.

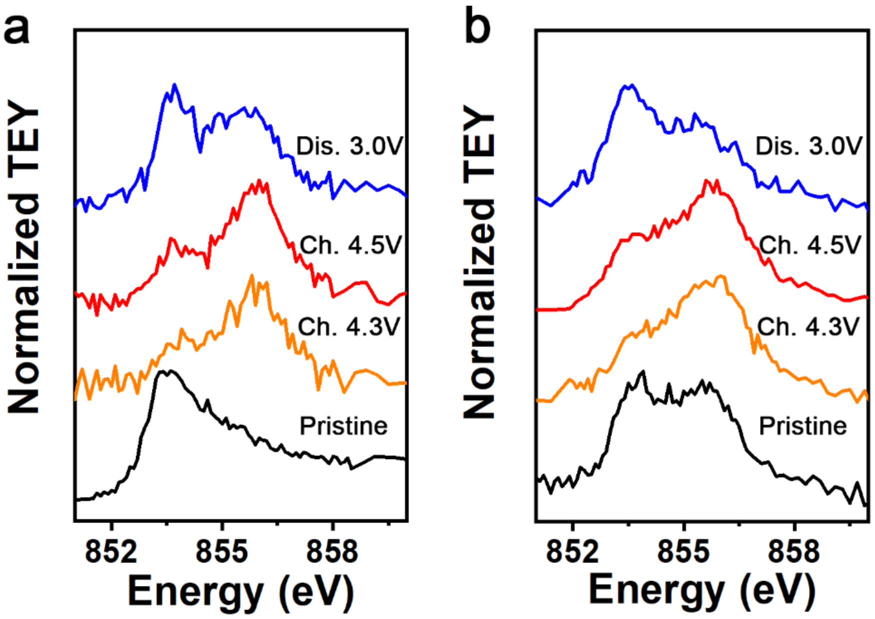

Figure S27. The soft-XAS spectra for 811-A (a) and 811-D (b) electrodes.

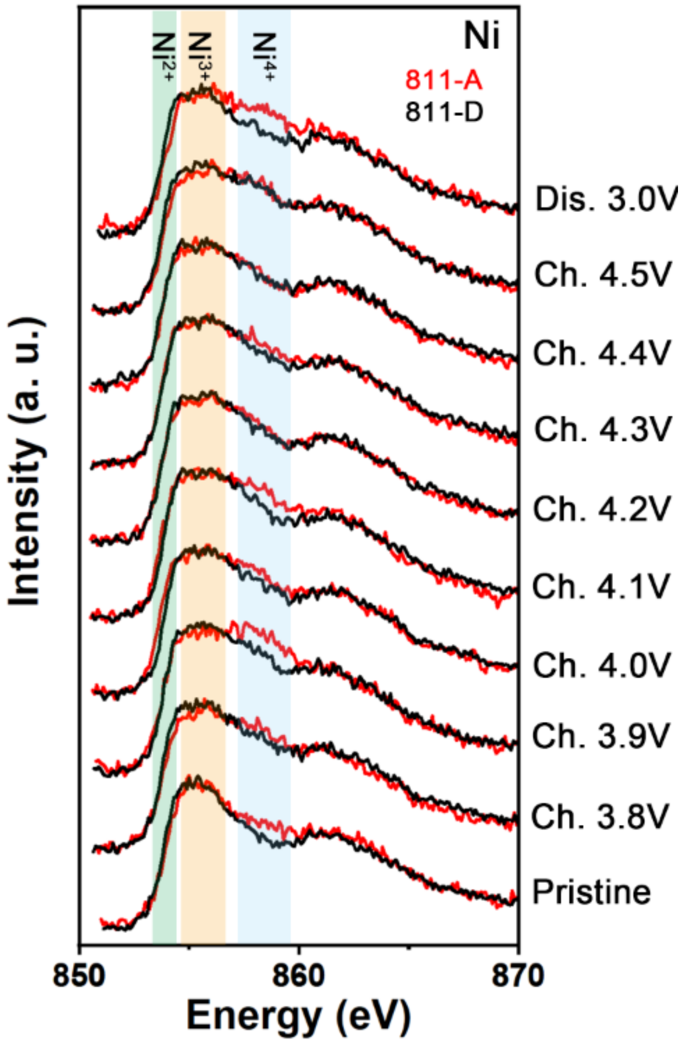

Figure S28. The Ni 2*p* XPS spectra for 811-A and 811-D electrodes.

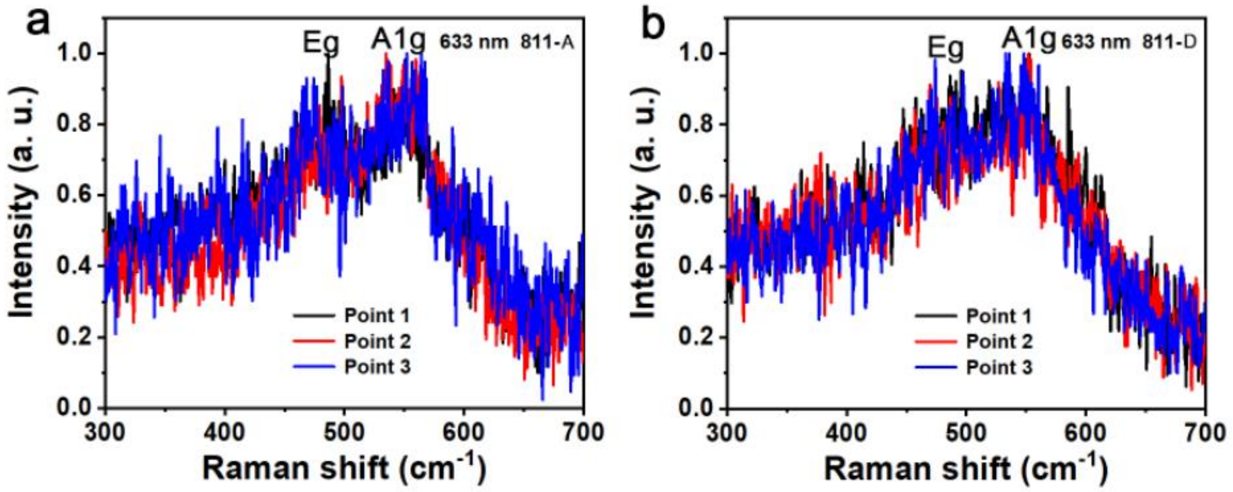

Figure S29. The Raman spectra for pristine 811-A electrode (c) and 811-D electrode (d) with a 633 laser.

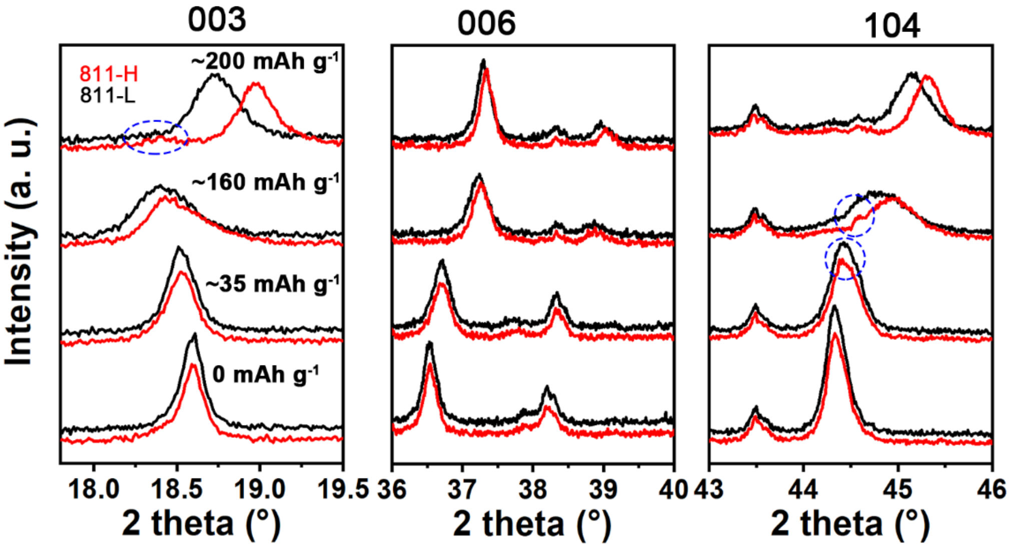

Figure S30. The picked XRD profiles tested at capacities of 0, ~35, ~160, ~200 mAh g^{-1}.

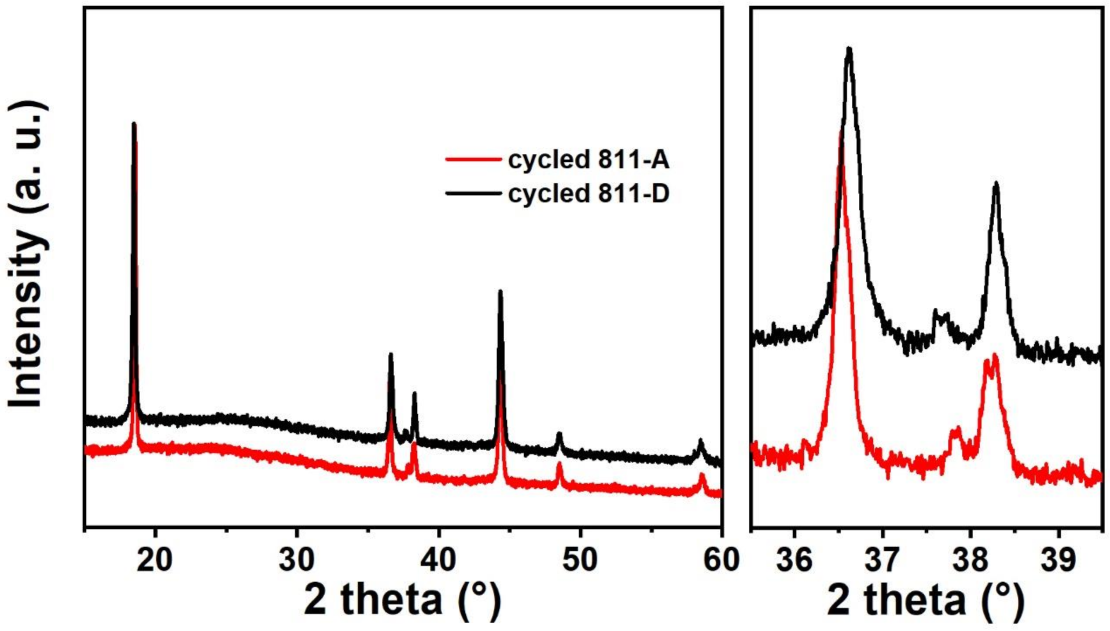

Figure S31. The XRD plots for 200 cycled electrodes at 1 C.

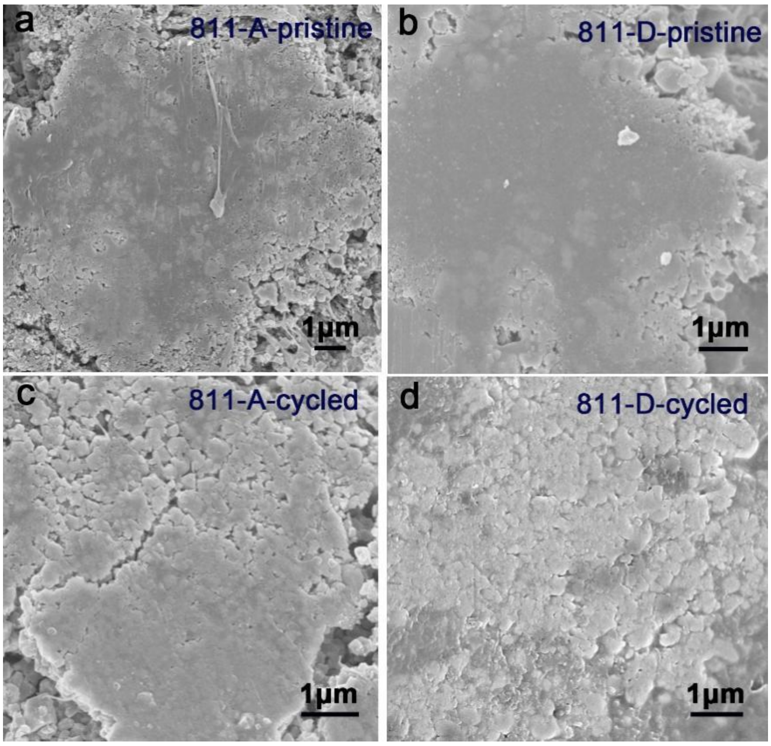

Figure S32. The cross-sectional SEM images for pristine 811-A electrode (a), pristine 811-D electrode (b), charge-discharged 811-A electrode with 80 cycles (c), charge-discharged 811-D electrode with 80 cycles (d).

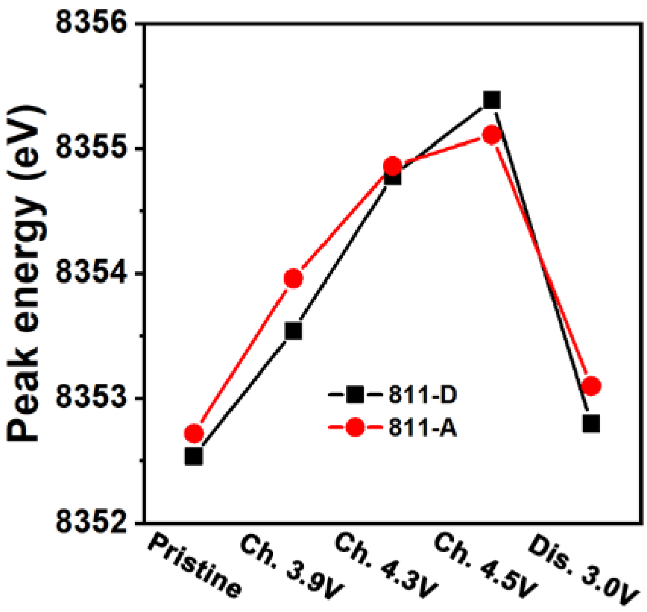

Figure S33. The white line peak positions in XAS spectra for 811-A and 811-D at different states of charge.

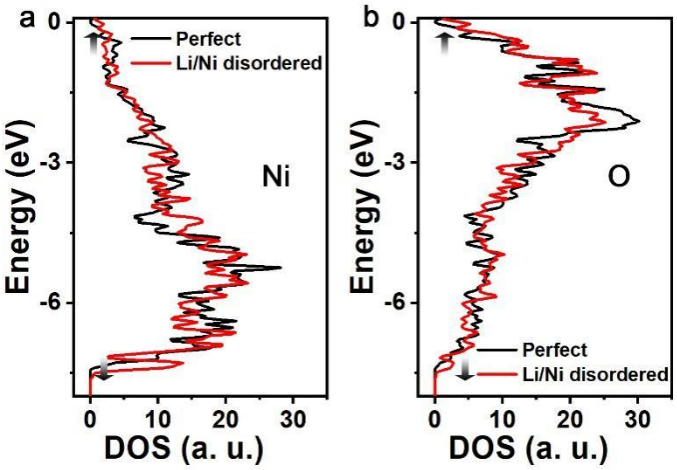

Figure S34. The white line peak positions in XAS spectra for 811-A and 811-D at different states of charge.

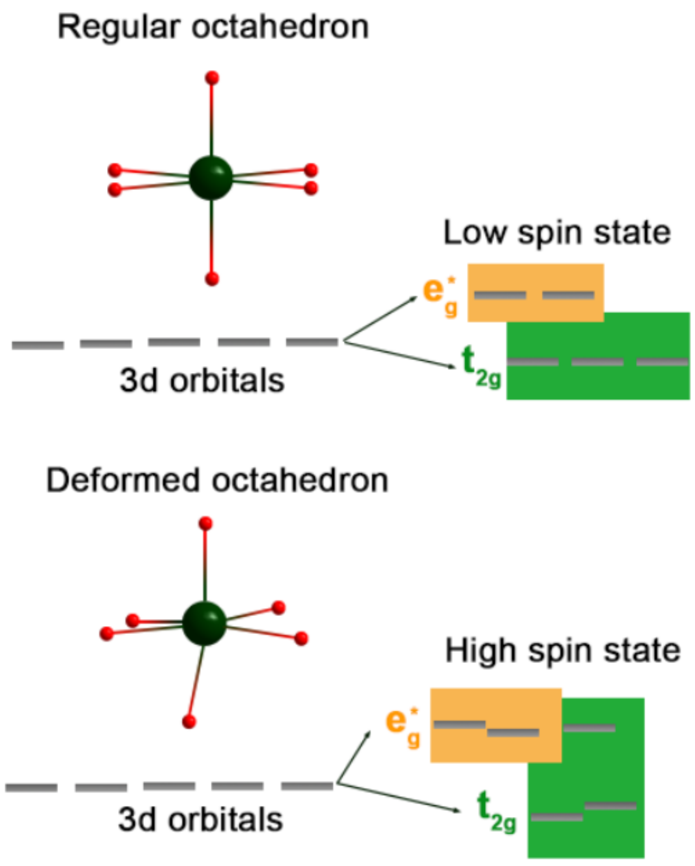

Figure S35. Sketch of orbital splitting in regular and deformed TMO_6 octahedrons.

Table S1. Elemental ratio of 811-A and 811-D derived from ICP-OES.

Samples	Ni	Co	Mn
811-A	0.8112	0.1024	0.0864
811-D	0.8103	0.1025	0.0872

Table S2. Cell parameters, Li/Ni disorder derived from XRD Rietveld refinement.

Samples	a [Å]	c (Å) [Å]	z_{ox}
811-A	2.8682(1)	14.1778(4)	0.2437(1)
811-D	2.8700(1)	14.1854(4)	0.2413(1)

Table S3. Cell parameters, Li/Ni disorder of 811-A derived from neutron diffraction Rietveld refinement.

a = 2.8616(1) Å, c = 14.1520(3) Å, Rwp = 4.749%					
Atoms	Site	x	y	z	Occupancy
Li	3a	0	0	0.5	0.9495(13)
Ni	3a	0	0	0.5	0.0504(12)
Ni	3b	0	0	0	0.7608(12)
Co	3b	0	0	0	0.1013(9)
Mn	3b	0	0	0	0.0894(5)
Li	3b	0	0	0	0.0505(13)
O	6c	0	0	0.25898(7)	0.9617(22)

Table S4. Cell parameters, Li/Ni disorder of 811-D derived from neutron diffraction Rietveld refinement.

a = 2.8701(1) Å, *c* = 14.1795(2) Å, *R*wp =2.680%					
Atoms	Site	*x*	*y*	*z*	Occupancy
Li	3a	0	0	0.5	0.9761(4)
Ni	3a	0	0	0.5	0.0234(8)
Ni	3b	0	0	0	0.7869(8)
Co	3b	0	0	0	0.1025(4)
Mn	3b	0	0	0	0.0874(24)
Li	3b	0	0	0	0.0239(4)
O	6c	0	0	0.25885(4)	1.0039(25)
